# The ZAT14 family promotes cell death and regulates expansins to affect xylem formation and salt tolerance in Arabidopsis

**DOI:** 10.1093/plcell/koaf271

**Published:** 2025-11-13

**Authors:** Ming Feng, Amrit K Nanda, Frauke Augstein, Ai Zhang, Lihua Zhao, Nilam Malankar, Sam W van Es, Bernhard Blob, Shamik Mazumdar, Jung-Ok Heo, Pawel Roszak, Jinbo Hu, Yrjö Helariutta, Charles W Melnyk

**Affiliations:** Department of Plant Biology, Swedish University of Agricultural Sciences, Almas allé 5, Uppsala 756 51, Sweden; College of Horticulture Science and Engineering, Shandong Agricultural University, Tai’an, 271018, China; Shandong Key Laboratory of Fruit and Vegetable Germplasm Innovation and Utilization, Shandong Agricultural University, Tai’an, 271018, China; Department of Plant Biology, Swedish University of Agricultural Sciences, Almas allé 5, Uppsala 756 51, Sweden; Department of Plant Biology, Swedish University of Agricultural Sciences, Almas allé 5, Uppsala 756 51, Sweden; Department of Plant Biology, Swedish University of Agricultural Sciences, Almas allé 5, Uppsala 756 51, Sweden; Peking University Institute of Advanced Agricultural Sciences, Shandong Laboratory of Advanced Agricultural Sciences in Weifang, Weifang, 261325, China; Department of Plant Biology, Swedish University of Agricultural Sciences, Almas allé 5, Uppsala 756 51, Sweden; Department of Plant Biology, Swedish University of Agricultural Sciences, Almas allé 5, Uppsala 756 51, Sweden; The Sainsbury Laboratory, University of Cambridge, Cambridge, CB2 1LR, UK; Department of Plant Biology, Swedish University of Agricultural Sciences, Almas allé 5, Uppsala 756 51, Sweden; The Sainsbury Laboratory, University of Cambridge, Cambridge, CB2 1LR, UK; The Sainsbury Laboratory, University of Cambridge, Cambridge, CB2 1LR, UK; Department of Plant Biology, Swedish University of Agricultural Sciences, Almas allé 5, Uppsala 756 51, Sweden; The Sainsbury Laboratory, University of Cambridge, Cambridge, CB2 1LR, UK; Institute of Biotechnology, Organismal and Evolutionary Biology Research Programme, Faculty of Biological and Environmental Sciences, Viikki Plant Science Centre, University of Helsinki, Helsinki, 00014, Finland; Department of Plant Biology, Swedish University of Agricultural Sciences, Almas allé 5, Uppsala 756 51, Sweden

## Abstract

The ability for stress to modify development is common in plants; yet, how external cues determine phenotypic outputs and developmental responses is not fully understood. Here, we uncovered a *ZINC FINGER OF ARABIDOPSIS THALIANA14* (*ZAT14*) transcription factor whose expression was enhanced in differentiating xylem through its positive regulation by *VASCULAR RELATED NAC-DOMAIN PROTEIN7* (*VND7*), yet, decreased in root tips through its negative regulation by *PLETHORA2* (*PLT2*) in Arabidopsis (*Arabidopsis thaliana*). Mutating *ZAT14* and its closely related homologs, *ZAT5*, *ZAT14L,* and *ZAT15*, disrupted vascular patterning and inhibited xylem differentiation indicating that ZATs are important for xylem formation. A transcriptome analysis of *zat* triple and quadruple mutants found that many cell wall-related genes were differentially expressed. In particular, 10 expansin genes were repressed by ZATs and several were direct targets of the ZATs. We uncovered that salinity repressed *ZAT14*, *ZAT14L*, and *ZAT15* vascular expression, whereas *zat* mutants improved salinity tolerance, decreased xylem differentiation, and reduced cell death mediated by salt. Furthermore, expansin mutants decreased salinity tolerance and increased xylem differentiation under salinity stress. We propose that ZATs are key regulators of programmed cell death that promote xylem formation, yet upon salinity stress, ZATs are repressed to inhibit cell death and improve salt tolerance, thus modifying developmental outputs in response to stress.

## Teaser

A family of ZAT14-related transcription factors are salt-responsive and enhance cell death and regulate expansin genes to promote xylem differentiation and affect salinity tolerance in Arabidopsis.

## Introduction

In vascular plants, xylem is fundamental for the transport of water and solutes from the roots to aerial tissues and plays a critical role in supporting plant growth and providing structural integrity (Hacke and Sperry 2001; De Boer and Volkov 2003). Its development and function are important for plant survival but also for adapting to environmental challenges (Ramachandran et al. 2021; Augstein and Carlsbecker 2022). Xylem development involves a tightly regulated process of secondary cell wall formation, programmed cell death, and lignification governed by various transcription factors. Particularly important are the *VASCULAR RELATED NAC-DOMAIN PROTEINs* (*VNDs*) expressed in differentiating vascular tissues that orchestrate downstream signaling pathways driving lignification, secondary cell wall formation, and cell death (Yamaguchi et al. 2008, 2010; Ohashi-Ito et al. 2010; Bollhöner et al. 2012). During such xylem differentiation, plant cells transition from producing primary cell walls (PCWs) to secondary cell walls (SCWs). In Arabidopsis, xylem differentiates in the root central vascular cylinder through an axis. The outer cells of xylem axis differentiate first to protoxylem with helical secondary cell wall structures. Later, cells of the inner xylem axis differentiate to metaxylem with pitted secondary cell wall structures. Such shapes and structures are thought to influence hydraulic conductance (Tang et al. 2018). VNDs are particularly important for activating SCW-related genes with *VND7* promoting protoxylem formation and *VND6* promoting metaxylem formation (Kubo et al. 2005; Ramachandran et al. 2021). Developing xylem accumulates lignin in addition to cellulose, hemicellulose, and pectin (Cosgrove 2005). Cellulose, a major constituent of both PCWs and SCWs, relies on primary and secondary cellulose synthase (CESA) genes for the formation of cellulose-synthesizing complexes (Kumar and Turner 2015; Li et al. 2016). Expansin proteins are also important for cell wall formation, and they disrupt hydrogen bonds between cellulose microfibrils to loosen and soften cell walls (Cosgrove 2000; Bashline et al. 2014). Expansins are stress responsive and induced by various hormones including auxin and abscisic acid (ABA) (Zhao et al. 2012), suggesting they might link environmental changes to cell wall modifications.

The ability of plants to modify their development in response to stress enables them to survive and thrive in diverse and changing environments. Xylem development is particularly responsive to stress. In response to drought, xylem morphology in *Arabidopsis* roots is modified so that extra protoxylem strands are formed and metaxylem differentiates closer to the root tip (Ramachandran et al. 2018). ABA also increases upon drought stress to activate VNDs that enhance xylem differentiation rates and convert metaxylem cells to protoxylem cells (Ramachandran et al. 2021). Tolerance to another stress, salinity, is thought to depend on avoidance strategies and the restriction of salt ion uptake and transport (Møller et al. 2009). Salinity stress causes cell death and affects vascular formation by inhibiting protoxylem differentiation to form protoxylem gap cells (PGC) that fail to undergo programed cell death. Such xylem gaps potentially reduce Na^+^ transport into the shoot and improving salt tolerance (Augstein and Carlsbecker 2022; Fedoreyeva et al. 2022). Salt induces ionic stress, osmotic stress, and the activation of ABA responses. ABA is important for osmotic stress responses; however, salinity-mediated changes in xylem differentiation do not appear ABA mediated but instead are regulated by gibberellic acid (Augstein and Carlsbecker 2022). Expansins have been implicated in salt stress response. Several expansins are upregulated by salt stress (Geng et al. 2013), and *EXPA1* mutants are resistant to salt-induced xylem gaps (Augstein and Carlsbecker 2022). In addition, overexpressing *EXPANSIN7* (*OsEXPA7*) increases salt stress tolerance in rice (Jadamba et al. 2020).

Multiple members of the C2H2-type zinc finger transcription factor family, including *AZF1*, *AZF2*, *ZAT5*, *ZAT6*, *ZAT7*, *ZAT10*, and *ZAT12*, respond to salinity and are related to programmed cell death (Sakamoto et al. 2004; Mittler et al. 2006; Ciftci-Yilmaz et al. 2007; Liu et al. 2013; Feng et al. 2023). *ZAT6*, *ZAT7*, *ZAT10,* and *AZF1/2* are induced by salinity treatment (Xie et al. 2019) whereas overexpression of *ZAT6* promotes salinity tolerance (Liu et al. 2013). A quintuple mutant of *ZAT14*, *AZF2*, *ZAT5*, *ZAT10*, and *ZAT12* delayed programmed cell death in *Arabidopsis* columella cell, while *ZAT14* overexpression promotes cell death and cell expansion (Roszak et al. 2021; Feng et al. 2023). Given the importance of cell death for xylem differentiation, and the role of xylem in salinity tolerance, it needs to be resolved how environmental stress such as salinity can regulate cell fate specification such as programmed cell death. Here, we focused on the transcription factor *ZAT14* (At5g03510), along with a group of closely related transcriptional factors *ZAT5* (At2G28200), *ZAT14-like* (At5g04390), and *ZAT15* (At3G10470), which we found were important for vascular differentiation downstream of VNDs. They showed rapid and strong differential expression under salinity stress. ZATs modified cell wall related processes, including repressing multiple expansin genes and contributed to enhancing salinity tolerance, thus balancing environmental stress tolerance with developmental of the vascular tissues.

## Results

### ZAT transcription factors express in developing xylem

**Figure 1.**
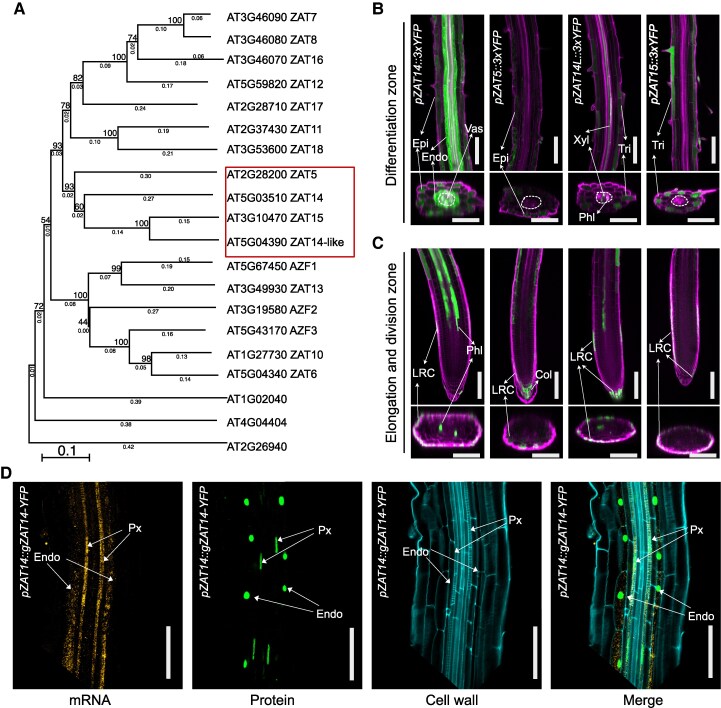
*ZAT*s are expressed in developing xylem. **A)** Phylogenetic tree of C1-2i subclass of the C2H2-type zinc finger protein family (20 genes). Bootstrap method with 1000 replicates amino acid with p-distance method, partial deletion with 50% site coverage cutoff. *ZAT14*, *ZAT14-like* (*ZAT14L*), *ZAT5*, and *ZAT15* belong to the same sub-clade, highlighted with a red rectangle. **B** and **C)** Expression patterns of *ZAT* transcriptional reporter lines in vascular tissue **(B)** and root tips **(C)**. The vascular tissue is marked in a circle. Green is YFP signal. Magenta is propidium iodide staining. Scale bars, 100 *µ*m in longitudinal sections and 50 *µ*m in cross sections. **D)** Whole-mount smFISH (WM-smFISH) on *pZAT14::gZAT14-YFP* showed mRNA (yellow) and protein (green) colocalized in developing protoxylem cells. mRNA molecules were detected with *VENUS* probes, cell walls were stained with Renaissance 2200. The cut off corner results from cropping. Scale bars, 50 *µ*m. All observations and treatments were used with five-day-old seedlings. The fluorescent cell types are annotated in the related images of (**B–D)**. Epi, epidermis; Endo, endodermis; Vas, vasculature; Phl, phloem; Xyl, xylem; Tri, trichoblast; LRC, lateral root cap; Col, columella; Px, protoxylem.

**Figure 2.**
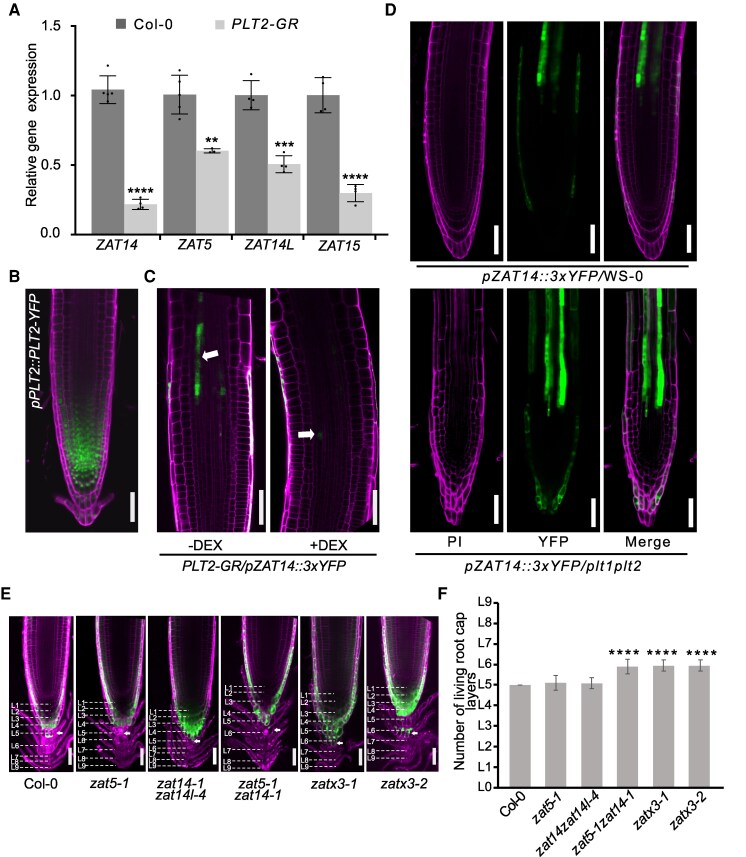
PLTs restrict *ZAT* expression levels. **A)** qRT-PCR of *ZAT* expression in *PLT2-GR* or Col-0. Five-day-old seedlings were treated with 10 *µ*M dexamethasone (DEX) for 24 h. Means ± SD from 4 to 5 replicates. Student's *t*-test compared with Col-0. **P < 0.01, ***P < 0.001, ****P < 0.0001. **B)** Expression pattern of *pPLT2::PLT2-YFP*, scale bars, 50 *µ*m. **C)** Confocal images showing *pZAT14::3xYFP* levels (green) in inducible *PLT2-GR* with or without 10 *µ*M DEX treatment for 24 h. White arrows indicate *ZAT14* fluorescence signal. Magenta is propidium iodide staining. The cut off corner results from cropping. Scale bars, 50 *µ*m. **D)**  *pZAT14::3xYFP* levels (green) in the wild type or *plt1plt2* mutant backgrounds. Magenta is propidium iodide staining. Scale bars, 50 *µ*m. Five-day-old seedlings were used for (**B–D)**. **E)** Living cell layers are stained with FDA in columella cells. Fourteen-day-old plants were used for staining, *n* = 13–20 roots per genotype. Magenta is propidium iodide staining. L means layers. Scale bars, 50 *µ*m. **F)** The quantification of the living root cap layers in (**E)**. Means ± SD. One-way ANOVA followed by Dunnett's multiple comparisons test compared with Col-0. ****P < 0.0001.

Previous work has found that *ZAT14* and its homologs *AZF2*, *ZAT5*, *ZAT10,* and *ZAT12* regulate cell death in root caps (Feng et al. 2023), so we sought to uncover whether ZAT transcription factors might also play a role in other cell types undergoing cell death. We investigated publicly available expression databases and found that *ZAT14*, as well as 3 closely related C2H2-type zinc finger protein family members from the C1-2i subclass, *ZAT5*, *ZAT14-like (ZAT14L),* and *ZAT15* (Fig. 1A), were all expressed in a similar pattern in the lateral root cap (LRC), phloem and xylem cells (Supplementary Fig. S1A) (https://rootcellatlas.org/). We generated transcription reporter lines for all 4 ZAT genes that showed signal in the differentiation zone and LRC cells but was absent from the division zone of the root apical meristem (Fig. 1, B and C). We further investigated the cellular localization of *ZAT14* using whole-mount smFISH assays with YFP and ZAT14 specific probes that were applied to *ZAT14* translational reporter line or wild type (Col-0), respectively. We found mRNA and protein of *ZAT14* colocalized in developing protoxylem cells (Fig. 1D; Supplementary Fig. S1B). Given that ZATs appeared to be excluded from the root division zone, we investigated their regulation and found that overexpressing the *PLETHORA2* (*PLT2*) transcription factor important for maintaining stem cell pools suppressed the expression of *ZAT5*, *ZAT14*, *ZAT14L,* and *ZAT15* (Fig. 2, A to C). On the other hand, the loss of function *plt1plt2* mutant increased *ZAT14* levels in the LRC and allowed a higher expression of *ZAT14* closer to the root tip (Fig. 2D). We analyzed previously published ChIP-seq data of PLT2 (Santuari et al. 2016) and found PLT2 directly bound the *ZAT5* locus but not to the *ZAT14*, *ZAT14L*, or *ZAT15* loci (Supplementary Fig. S2A). However, PLT2 also bound the promoters of *SMB* and *BRN2* (Supplementary Fig. S2A), 2 genes involved in programmed cell death in LRC and known to act upstream of *ZAT14* (Feng et al. 2023). PLT2 thus appeared to regulate these 4 ZATs both directly and indirectly. We next generated single, double, triple (*zatx3*), and quadruple (*zatx4*) mutant combinations of our ZATs of interest using T-DNA lines and CRISPR mutations (Supplementary Fig. S2, B and C). Given the high expression of ZATs in the LRC, we analyzed mutant phenotypes and found an additional, though weakly observed, living cell layer in the *zat5zat14* double mutant and a more pronounced layer in *zat* triple mutants (Fig. 2, E and F). These data were similar to previous findings using the quintuple *zat* mutant that included *ZAT5* and *ZAT14* mutations (Feng et al. 2023) but suggested that *ZAT5* and *ZAT14* were most relevant for LRC cell death (Fig. 2, E and F). Overall, it appeared that *ZAT5*, *ZAT14*, *ZAT14L,* and *ZAT15* expression was restricted to the developing xylem and LRC cells, and these genes played important roles in cell death.

### ZATs act downstream of VND7 to promote xylem differentiation

**Figure 3.**
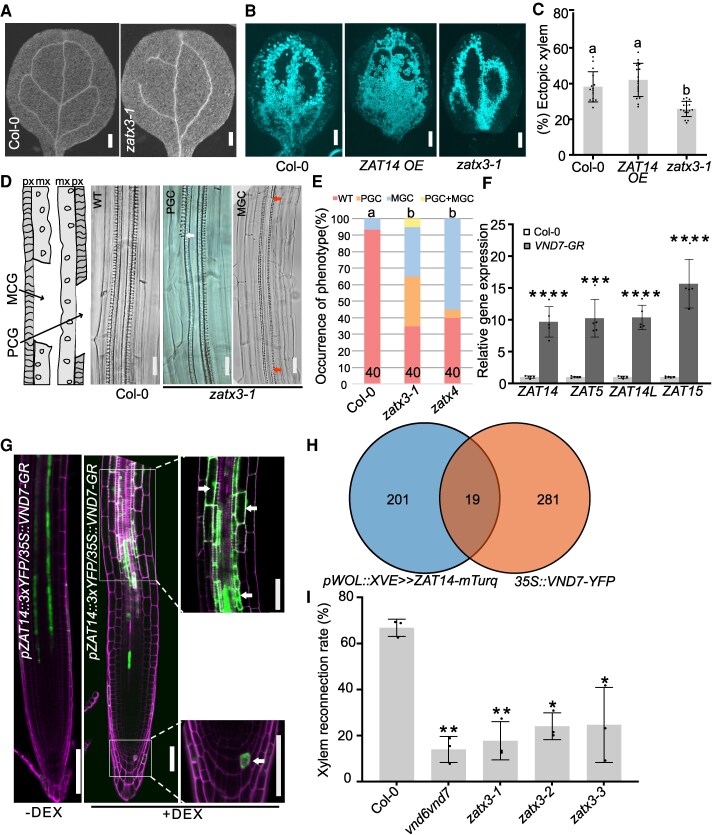
ZATs act downstream of VND7 to promote xylem differentiation. **A)**  *zatx3-1* mutants showed discontinuous vein vasculature in cotyledons of five-day-old seedlings. Scale bars, 100 *µ*m. **B)** Images of VISUAL treated cotyledons. Scale bars, 500 *µ*m. **C)** Quantifications of VISUAL xylem formation in Col-0 (*n* = 15), *ZAT14 OE* (*n* = 17) and *zatx3-1* (*n* = 17). Means ±SD. Letters indicate statistically significant differences. One-way ANOVA followed by Tukey's multiple comparisons test. **D)** Xylem gap cell phenotypes in *zatx3-1* mutants. Five-day-old seedlings were observed. The left cartoon panel shows xylem gap phenotypes. White arrow indicates PGC. Red arrow indicates MGC. Scale bars, 50 *µ*m. **E)** Quantifications of xylem gap cell phenotypes. Col-0 (*n* = 30), *zatx3-1* (*n* = 40), *zatx4* (*n* = 40). Means ± SD, one-way ANOVA followed by Tukey's multiple comparisons test. The statistical analysis was compared between WT and xylem gap (PGC, MGC, and PGC + MGC) phenotypes. **F)** qRT-PCR of *ZAT* expression 6 h after 10 *µ*M DEX induction with Col-0 and *VND7-GR*. Means ± SD from 5 replicates per genotype per treatment. ***P < 0.001, ****P < 0.0001. Two-tailed Student's *t*-test compared to Col-0. **G)**  *pZAT14::3xYFP* levels (green) in *35S::VND7-GR* with or without 10 *µ*M DEX induction for 24 h using five-day-old seedlings. White arrows indicate ectopic expression of *ZAT14* in cells with thick secondary cell wall. The cut off corner results from cropping. Scale bars, 100 *µ*m for the left of 2 images, 50 *µ*m for magnified new images of the same area. Magenta is propidium iodide staining. **H)** Venn diagram showing overlapping upregulated genes between *pWOL::XVE>>ZAT14-mTurq* (data adapted from Roszak et al. 2021) and *35S::VND7-YFP* (data adapted from Yamaguchi et al. 2011). **I)** Xylem reconnection rates of *vnd6vnd7*, *zat3-1*, *zatx3-2*, *zatx3-3* at 5 days after grafting. Three replicates with 10–12 plants per genotype per treatment. Means ± SD. One-way ANOVA followed by Dunnett's multiple comparisons test compared with Col-0. *P < 0.05, **P < 0.01. px, protoxylem; mx, metaxylem; WT, wild type xylem phenotype; PGC, protoxylem gap cells; MGC, metaxylem gap cells.

**Figure 4.**
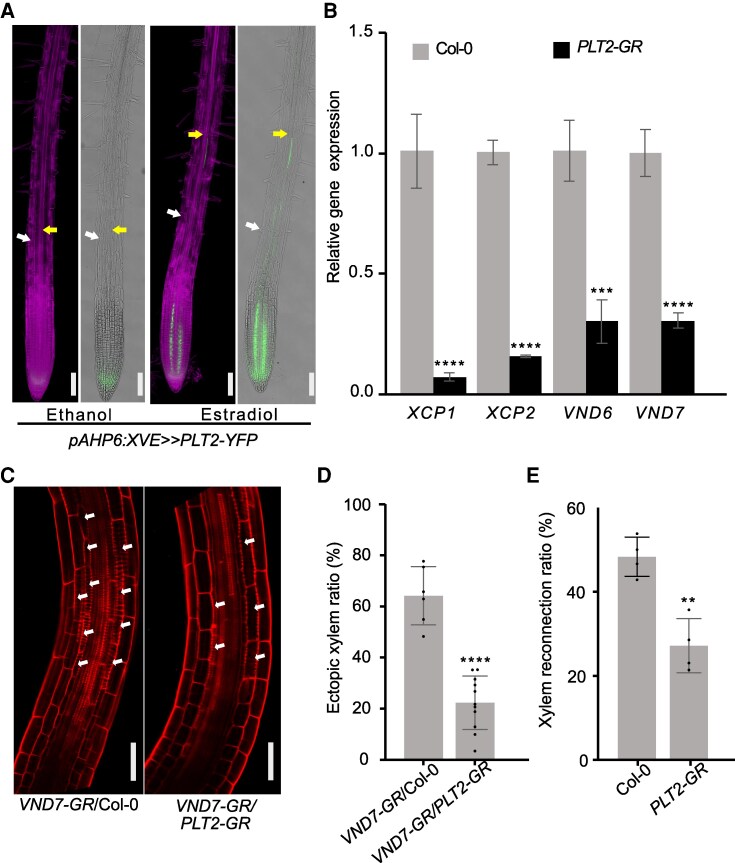
PLT2 inhibits VND7-induced xylem formation. **A)**  *pAHP6::XVE>>PLT2-YFP* expression delays xylem differentiation. White arrows indicate the first root hair. Yellow arrows indicate the start of differentiated xylem. Five-day-old seedlings were treated with 10 *μ*M estradiol for 24 h, ethanol treatment was as control. Magenta is propidium iodide staining. *PLT2-YFP* green signal is in the nuclear. Scale bars, 100 *µ*m. **B)**  *PLT2-GR* induction repressed xylem-related genes expression in five-day-old seedlings. Means ± SD. ***P < 0.001, ****P < 0.001. Twenty-four-hour induction with 10 *µ*M DEX. **C)**  *VND7-GR* induced ectopic xylem in the *PLT2-GR* or wildtype background. Images taken 24 h after induction with 20 µM DEX in five-day-old seedlings. Red is propidium iodide staining. White arrows indicate ectopic xylem. The cut off corner results from cropping. Scale bars, 50 *µ*m. **D)** Quantification of the ectopic xylem ratio. The quantified area is from the first elongated cell upwards to the fifth cell in the elongation zone. *VND7-GR*/Col-0 (*n* = 6), *VND7-GR/PLT2-GR* (*n* = 11). Mean ± SD. Two-tailed student's *t*-test. ****P < 0.0001. **E)** Xylem reconnection rates 5 days after grafting in wild type or *PLT2-GR* backgrounds. 20 *µ*M DEX or equal volume of EtOH were treated on plants for 4 hours before grafting, and 20 *µ*M DEX or equal volume of EtOH were used instead of water in the grafting setup. Means ± SD from 4 replicates. Student's *t*-test compared with Col-0. **P < 0.01.

Given the expression of *ZAT5*, *ZAT14*, *ZAT14L,* and *ZAT15* in the vascular tissues, we first analyzed single and higher order *zat* mutants for cotyledon vascular patterns and found *zat* mutants failed to form closed vein vascular loops (Fig. 3A and Supplementary Fig. S3A). We used the Vascular Cell Induction Culture System using *Arabidopsis* Leaves (VISUAL) assay to examine ectopic xylem formation (Kondo et al. 2016) and found significant decreases in ectopic xylem formation in *zat* mutants and a slight but insignificant increase in a previously published *ZAT14* over expression (*ZAT14 OE*) line (Fig. 3, B and C) (Feng et al. 2023). In roots, *zat* mutants increased protoxylem gap cells (PGC) and metaxylem gap cells (MGC) where xylem elements failed to differentiate, suggesting that ZATs affect xylem cell death (Fig. 3, D and E). *VASCULAR-RELATED NAC-DOMAIN7* (*VND7*) acts as a key regulator of xylem vessel differentiation and overexpressing *VND7-SRDX* or mutating *VND6* and *VND7* leads to discontinuous formation of xylem (Kubo et al. 2005; von der Mark et al. 2022). Given the similarity of phenotypes between *vnd* and *zat* mutants, we investigated whether xylem gap formation in *zat* mutants was mediated by *VND7*. An inducible *VND7-GR* line upregulated *ZAT5*, *ZAT14*, *ZAT14L,* and *ZAT15* (Fig. 3F) whereas the *ZAT14* reporter showed ectopic expression outside of the vascular tissues upon induction of *VND7* (Fig. 3G). To look for genes upregulated by both *ZAT14* and *VND7*, we compared previously published *ZAT14* and *VND7* overexpression datasets (Yamaguchi et al. 2011; Roszak et al. 2021) and found 19 genes induced by both *ZAT14* and *VND7* (Fig. 3H). Notably, many of these genes were associated with cell wall formation, lignification and xylem differentiation (Supplementary Fig. S3, B and C). Plant grafting also involves xylem formation at the junction and in grafting assays we found that both *vnd6vnd7,* which exhibits discontinuous xylem cells (von der Mark et al. 2022), and *zat* mutants delayed xylem reconnection (Fig. 3I). *VND7-GR* induces ectopic xylem (Kubo et al. 2005) and such xylem formation was blocked in the vascular tissues of the *zatx3* mutant where ZATs express (Supplementary Fig. S3,D and E). Given the link between *PLT2* and ZATs, we investigated if *PLT2* was also involved in xylem differentiation. Using a previously published *pAHP6::XVE>>PLT2-YFP* line (Mähönen et al. 2014), where *PLT2* is expressed in the protoxylem and neighboring pericycle cells, we found upon induction that xylem differentiation was delayed (Fig. 4A). An inducible overexpression *PLT2-GR* line (Galinha et al. 2007) repressed key xylem-related genes *XCP1*, *XCP2*, *VND6*, and *VND7* (Fig. 4B). The *PLT2-GR* line also blocked ectopic xylem formation in the *VND7-GR* line and reduced xylem formation at the graft junction (Fig. 4, C to E). Our results suggested that *PLT2* and ZATs play an import role in xylem development acting either upstream or downstream, respectively, of *VND7* to mediate xylem differentiation.

### ZATs affect cell wall processes

**Figure 5.**
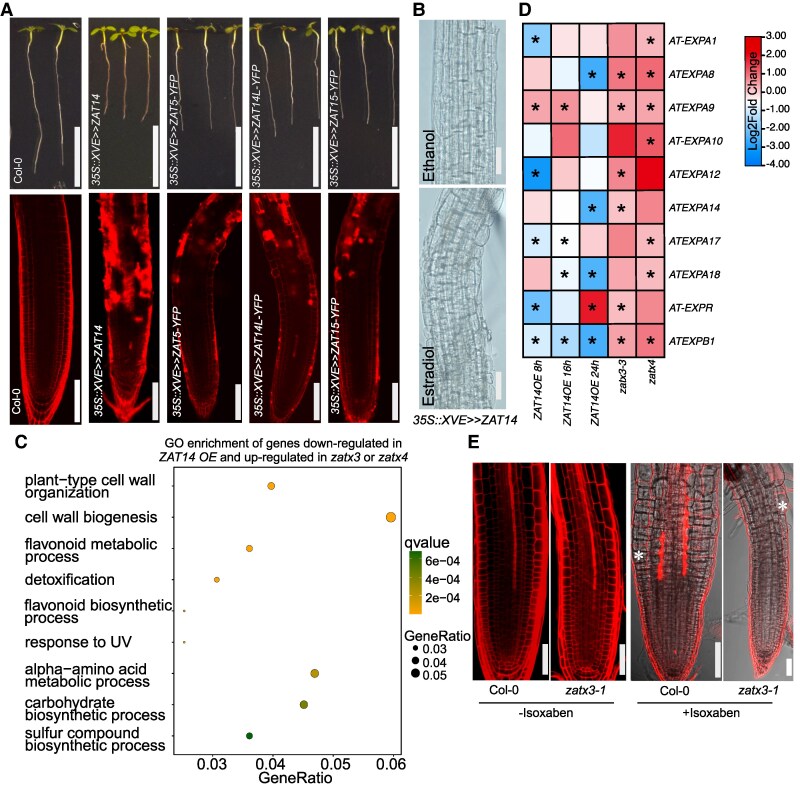
ZATs affect cell wall modification. **A)** Overexpressing *ZAT14*, *ZAT5*, *ZAT14L*, and *ZAT15* promotes cell wall damage. Upper panels, five-day-old seedlings were induced with 10 *µ*M estradiol for 48 h, scale bars, 1 cm. Lower panels, confocal images of roots induced with 10 *µ*M estradiol for 24 h. Roots were stained with propidium iodide (red). Scale bars, 50 *µ*m. **B)**  *35S::XVE>>ZAT14* promoted cell swelling. Five-day-old seedlings were induced with ethanol or estradiol (5 *µ*M) for 24 h. Scale bars, 100 *µ*m. **C)** Gene ontology (GO)–enrichment analysis of overlapped genes downregulated in *35S::XVE>>ZAT14* (*ZAT14 OE*) but upregulated in *zat* mutants. **D)** Heatmap showing expansin genes regulated by ZATs. **E)** Five-day-old wild-type of *zat3x-1* mutant seedlings were treated with 10 nm isoxaben for 24 h, *n* = 10, scale bars, 50 *µ*m. Red is propidium iodide staining. No isoxaben-treated seedlings were used as controls on the left panels. The asterisks indicate the first of swelling cell.

To understand the role of ZATs in cell death, we took a previously published *ZAT14 OE* line and confirmed it caused cell death, similar to previous findings (Feng et al. 2023), and additionally found that over expressing *ZAT5*, *ZAT14L,* or *ZAT15* caused cell death, enhanced cell swelling, and inhibited root growth (Fig. 5, A and B, Supplementary Fig. S4A). Next, we generated RNA sequencing libraries of *ZAT14 OE* and *zat* mutants and found several thousand genes were differentially expressed. The *zatx3* mutant exhibited more differentially expressed genes than the *zatx4* mutant, consistent with the more severe xylem defects observed in *zatx3* mutants (Fig. 3E, Supplementary Figs. S3A and S4B and Supplementary Data Set 1). Since *ZAT14* has been identified as a transcriptional repressor (Feng et al. 2023), we focused on genes downregulated by *ZAT14 OE* and upregulated in *zatx3 or zatx4* mutants to identify 568 potential target genes of *ZAT14* (Supplementary Data Set 2). A gene ontology (GO) analysis revealed enrichment of cell wall organization and cell wall biogenesis related genes (Fig. 5C). Among the genes regulated by *ZAT14* (Supplementary Data Set 2), we found 10 members of the cell-wall loosening expansin family were upregulated in *zat* mutants suggesting expansins might act downstream of ZATs (Fig. 5D, Supplementary Data Set 3). Consistent with a role in cell wall homeostasis, *ZAT14 OE* increased lignification and cell swelling (Fig. 5B, Supplementary Fig. S4C). Such phenotypes were also similar to chemical treatments with the primary cellulose biosynthesis inhibitor isoxaben (Denness et al. 2011; Basu and Haswell 2020). Moreover, several primary and secondary CESA genes were downregulated in *ZAT14 OE* and slightly, but insignificantly, upregulated in the *zat* mutants (Supplementary Fig. S4D). The *zatx3* mutant showed no major differences in cell size compared to Col-0 but had a smaller meristem zone (Fig. 5E). However, following isoxaben treatment, the *zatx3* mutant was resistant to isoxaben-induced cell swelling and cell death (Fig. 5E). Given that PLT2 repressed ZATs expression, we also observed that PLT2 repressed *ZAT14-*mediated or isoxaben-induced cell death and cell swelling (Supplementary Fig. S4, E to G). Together, these results indicated that ZATs acted in part by modifying cell wall–related processes.

### Salinity-repressed ZATs target expansin genes

**Figure 6.**
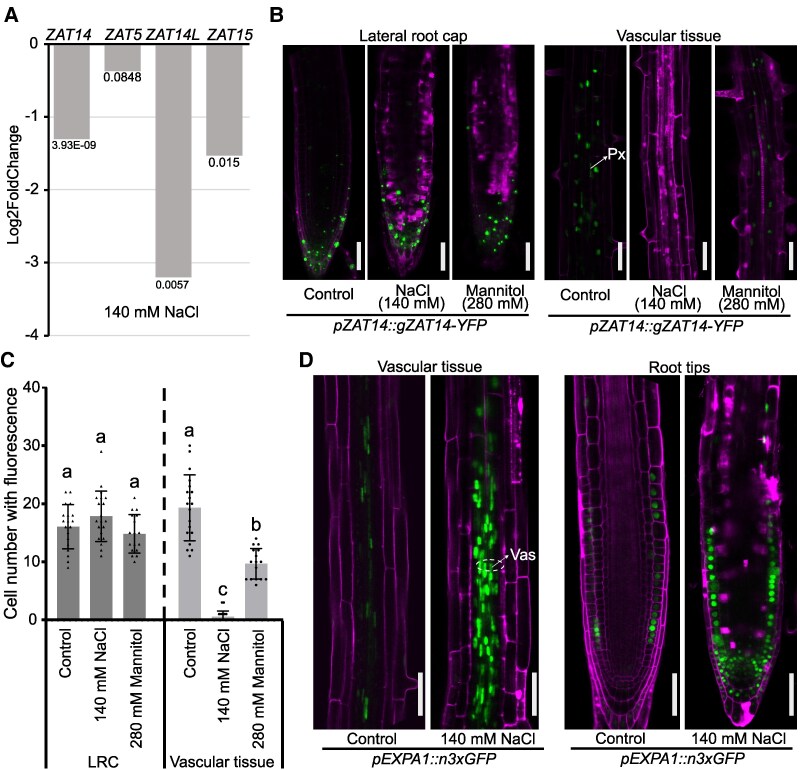
Salinity downregulates *ZAT* expression and upregulates EXPAs. **A)** RNA-Seq data of *ZAT* expression after 1 h salt treatment, data taken from (Augstein and Carlsbecker 2022). *P*-value is shown. **B)**  *pZAT14::gZAT14-YFP* levels (green) in response to salt (140 mm NaCl) or mannitol (280 mm) treatment for 24 h in lateral root cap (LRC) or vascular tissues. Magenta is propidium iodide staining. Px, protoxylem. The cut off corner results from cropping. Scale bars, 50 *µ*m. **C)** Quantifications of fluorescence signal in **B**. Control *n* = 19; salt *n* = 20; mannitol *n* = 17. Mean ± SD. Letters indicate statistically significant differences. One-way ANOVA followed by Tukey's multiple comparisons test. **D)** Salt increases *pEXPA1::n3xGFP* levels (green), *n* means nuclear localization signal. Images were taken 24 h after salt treatment. Magenta is propidium iodide staining. Vas, vascular. Scale bars, 50 *µ*m.

**Figure 7.**
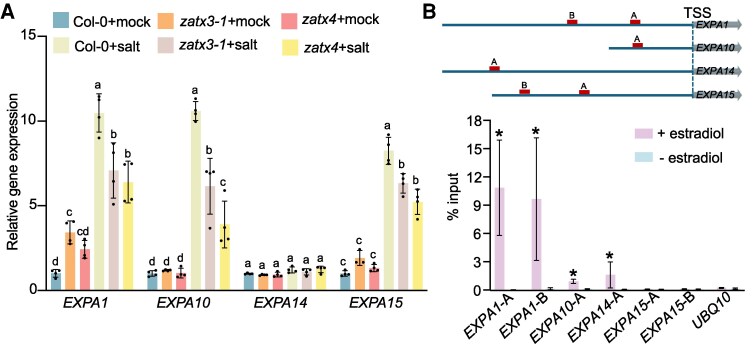
EXPAs act downstream of ZAT14. **A)** qRT-PCR results showed *EXPAs* expression in Col-0 and *zat* mutants with or without salinity treatment for 1 h. Five-day-old seedlings were used for experiments. Letters indicate statistically significant differences. One-way ANOVA followed by Tukey's multiple comparisons test. **B)** ChIP-qPCR showed ZAT14 directly binds the promoter of *EXPA1*, *EXPA10* and *EXPA14*. Schematic representation of genes showing the characterized binding regions on their promoters. *UBQ10* was used as a negative control. Three replicates. Means ± SE. The Mann–Whitney *U* test was used for statistical differences, *P < 0.05. TSS, transcription start site.

Previous work has shown that inhibiting xylem differentiation is important for salinity tolerance (Augstein and Carlsbecker 2022), and our data showed that ZATs affect xylem differentiation (Fig. 3, D and E), so we tested whether ZATs had a function in salt stress. We took a previously published RNAseq dataset of salinity treatment in roots (Augstein and Carlsbecker 2022) and observed that salinity repressed *ZAT14, ZAT14L*, and *ZAT15* expression levels (Fig. 6A). We confirmed these findings with the *ZAT14* translational reporter and found the effect was specific to the vascular tissues and not the LRC 16 h and 24 h after salinity treatment (Fig. 6, B and C, Supplementary Fig. S5B). We next investigated the salt response of the *ZAT* transcriptional reporters and found that salinity repressed *ZAT14* and *ZAT14L* expression in vascular tissues, whereas salinity increased *ZAT14L* and *ZAT15* expression in epidermal layers (Supplementary Fig. S5A). We also observed that mannitol slightly decreased *ZAT14* fluorescence suggesting that the osmotic stress aspect of salt treatment played a minor role in *ZAT14* reduction (Fig. 6, B and C). We next compared genes differentially expressed by ZATs with those differentially expressed by salt and found a substantial enrichment in cell wall–related processes comparing common genes downregulated by ZATs but upregulated by salt (Supplementary Fig. S6, A and B, Supplementary Data Set 4). Given that ZATs repressed expansin genes (Fig. 5), and the established role of EXPA1 in salinity-induced xylem differentiation (Augstein and Carlsbecker 2022), we tested an *EXPA1* reporter and found increased signal in the vascular cells and LRC upon salinity treatment (Fig. 6D). However, *ZAT14* and *EXPA1* expression appeared to overlap primarily in the stele and xylem (Fig. 1C, Fig. 6D, Supplemental Figs. S5C). To further investigate how ZATs regulate expansins, we performed qRT-PCRs and found salinity increased the expression of *EXPA1*, *EXPA10*, and *EXPA15*. Under mock conditions, *EXPA1* expression was significantly higher in *zat* mutants compared to Col-0; however, when treated with salt, *EXPA1*, *EXPA10*, and *EXPA15* expression was lower in *zat* mutants compared to Col-0 (Fig. 7A), suggesting ZATs can promote EXPA expression during salinity stress. ChIP-qPCR assays showed that ZAT14-YFP bound the promoters of *EXPA1*, *EXPA10*, and *EXPA14* (Fig. 7B, Supplementary Fig. S7A). We also found that ZAT14 bound the promoter of *PRX71* (Supplementary Fig. S7A), a cell wall–bound peroxidase gene involved in lignin structure regulation (Shigeto et al. 2013). *PRX71* expression was downregulated in *ZAT14* overexpression lines and upregulated in *zat* mutants (Supplementary Fig. S7B), consistent with *PRX71* being another direct target of ZAT14. These results indicate that ZATs are repressed by salinity whereas expanins are upregulated by salinity, and that ZATs play a major role in binding to and repressing expansin expression.

### ZATs promote salt sensitivity by enhancing cell death

**Figure 8.**
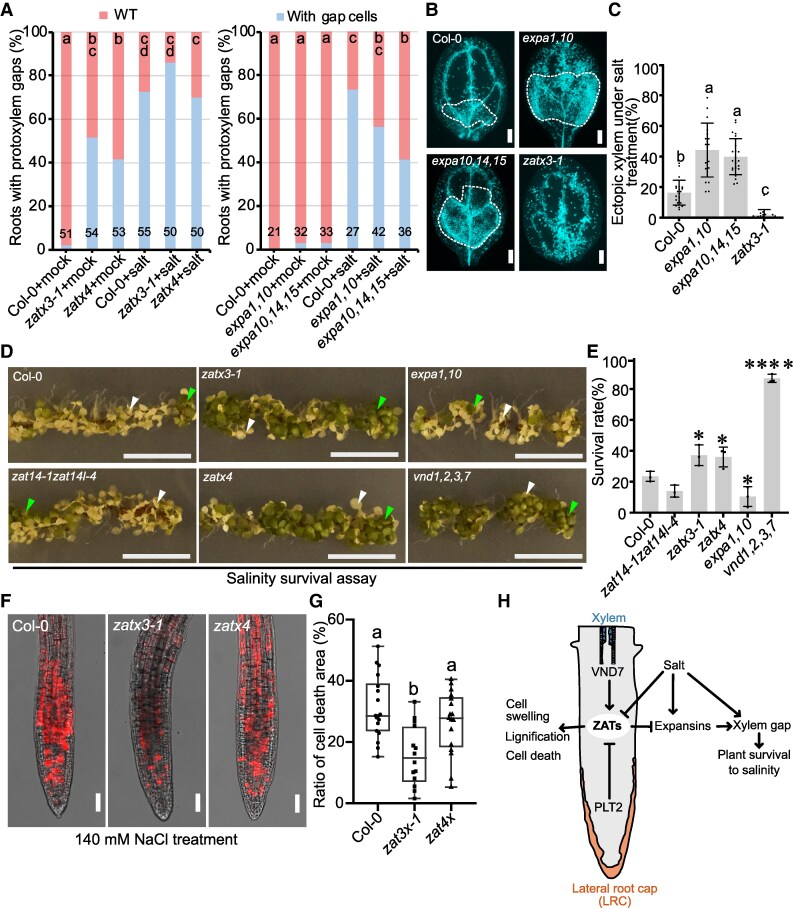
ZATs promote salt tolerance primarily through expansion-mediated effect on xylem development. **A)** Xylem gap phenotype after salt treatment. Three-day-old seedlings were treated with 140 mm NaCl for 3 d. Seedling numbers used for quantification were shown in the plots; letters indicate statistical significance with multiple Fisher's exact test and Benjamini–Hochberg (BH) correction. **B)** VISUAL assay using 100 mm NaCl in the induction solution. Scale bars, 500 *µ*m. The induced ectopic xylem area is outlined within white dash line. **C)** Quantification of ectopic xylem area in **B**. Col-0 (*n* = 22), *expa1,10* (*n* = 19), *expa10,14,15* (*n* = 25), *zatx3-1* (*n* = 17). Means ± SD, one-way ANOVA followed by Tukey's multiple comparisons test. Different letters represent *P* < 0.05. **D)** Salinity tolerance of mutants. Three-day-old seedlings were transferred to medium with NaCl (200 mm) for 5 d. Surviving seedlings were highlighted with green triangles and dead seedlings with white triangles. Scale bars, 1 cm. **E)** Survival rate of plants on 200 mm salt. Means ± SD, 3 replicates with Col-0 (*n* = 173), *zat14-1zat14l-4* (*n* = 153), *zatx3-1* (*n* = 149), *zatx4* (*n* = 137), *expa1,10* (*n* = 136), *vnd1,2,3,7* (*n* = 109). One-way ANOVA followed by Dunnett's multiple comparisons test compared with Col-0. *P < 0.05, ****P < 0.0001. **F)** Salinity-induced cell death in root tips. Five-day-old seedlings treated with NaCl (140 mm) for 4 h. Scale bars, 50 *µ*m. **G)** Quantification of cell death area in **F)**. One-way ANOVA followed by Tukey's multiple comparisons test. Col-0 (*n* = 18), *zatx3-1* (*n* = 15), *zatx4* (*n* = 17). **H)** Hypothetical model for ZATs regulating xylem differentiation and salt tolerance. ZATs are positive regulated by *VND7*, yet, decreased in root tips through their negative regulation by *PLT2*. Salinity repressed *ZATs* vascular expression but upregulated *EXPAs* expression to promote xylem gap formation, whereas xylem gaps improved salt tolerance. *ZAT* overexpression promoted cell swelling, lignification, and cell death.

Given their regulation by salt, we tested *ZAT* mutants for salt-induced phenotypes and observed that *zat* triple and quadruple mutants increased protoxylem gap numbers under mock conditions compared to Col-0 but showed a similar number of gaps as Col-0 under salt conditions (Fig. 8A). *EXPA10*, *EXPA14*, and *EXPA15* show functional redundancy with *EXPA1* (Pacifici et al. 2018), and we observed wild-type levels of protoxylem gaps in *expa1,10* and *expa10,14,15* mutants under mock conditions; however, salt treatments showed fewer xylem gaps in *expa* mutants compared to Col-0 (Fig. 8A). Similarly, an *EXPA1* over expressing line showed more xylem gaps with salt treatment compared to Col-0 (Supplementary Fig. S8A). These data suggested that ZATs promoted xylem differentiation, whereas expansins repressed xylem differentiation specifically under salinity. VISUAL assays under mock conditions showed no changes with expansin mutants, but upon salt treatment, *expa1,10* and *expa10,14,15* mutants increased xylem formation (Fig. 8, B and C, Supplementary Fig. S8, B and C). Additionally, *zatx3-1* exhibited a lower ectopic xylem area compared to Col-0 consistent with ZATs promoting xylem formation (Fig. 8, B and C). To assess the salinity tolerance of mutants, we counted the survival score or survival rate of plants subjected to lower (140 mm) or higher (200 mm) concentration of salt (Augstein and Carlsbecker 2022) and found *zat* triple and quadruple mutants showed higher survival scores and survival rates compared with Col-0 and *zat14-1zat14l-4* double mutants (Fig. 8, D and E, Supplementary Fig. S8, D and E). *vnd1,2,3,7* and *expa1,10* mutants demonstrated even higher and lower survival rates, respectively (Fig. 8, D and E, Supplementary Fig. S8, D and E). The *ZAT14 OE* line showed lower survival scores and fewer xylem gaps on salt, consistent with gaps correlating with salinity tolerance (Supplementary Fig. S8, F and G). Salinity delays xylem cell death yet promotes root tip cell death, so we tested *zat* mutants and found they showed less cell death in root tips under salt treatment compared to Col-0 (Fig. 8, F and G). Together, these results suggest that ZATs promoted salt sensitivity by promoting cell death.

## Discussion

### ZATs promote xylem differentiation

Here, we identified a group of 4 ZAT transcription factors that were important for xylem differentiation. Previous work has implicated *AZF2*, *ZAT5*, *ZAT10*, *ZAT12, ZAT14* in programmed cell death of the columella root tip cells (Feng et al. 2023) and here we demonstrated a critical role for ZATs in vascular programmed cell death. Triple mutants with *ZAT5*, *ZAT14,* and *ZAT14L* showed strong xylem phenotypes, whereas addition of *ZAT15* caused minor enhancements. However, *ZAT5* and *ZAT14* seemed most important for programmed cell death since their double mutant showed columella programmed cell death phenotypes and leaf venation phenotypes as strong or nearly as strong as the triple and quadruple mutants (Fig. 2F, Supplementary Fig. S3A). We found ZATs were expressed in 2 tissues undergoing programmed cell death, the lateral root cap and the xylem, but intriguingly, they were also expressed in phloem cells (Supplementary Fig. S1A). *ZAT14* and *ZAT14 l* are expressed in protophloem cells before enucleation when *APL* and *ANAC045/086* express (Furuta et al. 2014; Roszak et al. 2021). Although we only observed xylem vascular defects in *zat* mutants, it raises the intriguing possibility that ZATs are involved in a programmed cell death-like process involving cell wall changes and enucleation in the phloem. Our study also placed ZATs in a regulatory network comprising of VNDs and PLTs. PLTs have been previously implicated in repressing xylem identity in root tips (Mähönen et al. 2014; Santuari et al. 2016), and our data provide mechanistic insight to show that PLT2 repressed *ZAT*, *VND6/7,* and *XCP1/2* expression (Fig. 2, A to D, Fig. 4B), suggesting *PLT2* inhibits multiple points of the xylem differentiation pathway. The ability of *PLT2* to suppress such a pathway is likely a means to avoid vascular differentiation in the meristem. *VND7* also acted upstream of all 4 ZATs to promote their expression (Fig. 3F), and we found both protoxylem gap cells and metaxylem gap cells in *zat* mutants suggesting that ZAT regulation by VNDs is an important component promoting xylem differentiation during non-stressed conditions.

### ZATs affect the cell wall

Our GO analysis of *zat* mutant transcriptomes identified downstream target genes that were enriched in cell wall organization and biogenesis function. In particular, 10 members of the expansin gene family were upregulated in the *zat* triple and quadruple mutants suggesting that ZATs negatively regulated this gene family, consistent with the putative role for *ZAT14* as a transcriptional repressor (Feng et al. 2023). Previous studies have shown that expansins are abundant in xylem cells (Im et al. 2000; Gray-Mitsumune et al. 2004), and our findings showed that expansin mutants promoted xylem formation during salt stress. However, further work is needed to determine how expansins might repress xylem differentiation. Expansins are involved in cell wall loosening and interrupt the hydrogen bonds between cellulose microfibrils and cross-linking glycans within the cell wall (McQueen-Mason and Cosgrove 1994, 1995). Overexpressing *ZAT14* or *ZAT14 l* causes cell cycle arrest and cell elongation (Roszak et al. 2021), and we propose such swelling is mediated in part by cell wall modifications mediated by expansins. Notably, our *ZAT14 OE* line caused cell death, cell swelling, and lignification, which all phenocopy treatment with isoxaben, a chemical inhibitor of primary cellulose biosynthesis (Denness et al. 2011; Watanabe et al. 2018). In addition, our transcriptome analysis showed that *ZAT14OE* reduced the expression of primary CESAs (*CESA1*, *CESA3*, and *CESA5*) at early time points but not secondary CESAs (*CESA4*, *CESA7*, and *CESA8*) (Supplementary Fig. S4D). The early downregulation of primary CESAs suggests a transition toward secondary cell wall formation during xylem differentiation (Watanabe et al. 2018) and could hint at how ZATs are involved with xylem formation. Surprisingly, *zat* mutants were resistant to isoxaben-induced cell swelling. Isoxaben-resistant mutants often have defects in cell wall or cellulose synthesis, indicating that *zat* phenotypes might arise via modifications in the composition or properties of cellulose though primary CESA gene expression was slightly, but not significantly, increased in *zat* mutants (Supplementary Fig. S4D). Such cellulose and expansin-mediated modifications could be highly relevant for cell differentiation during programmed cell death, an aspect that warrants further investigation. *VND* overexpression causes multiple cell types to turn to xylem (Yamaguchi et al. 2010); yet, *ZAT* overexpression caused cells to die without obtaining xylem morphology, suggesting that ZATs might promote or prime an early stage of cell death before secondary cell wall formation. We also observed that ectopic xylem formation from *VND7* overexpression depended partly on ZAT function (Supplementary Fig. S3D) consistent with ZATs acting downstream of VNDs; yet there are likely additional factors downstream of VNDs that cause ectopic xylem formation.

### Salinity represses ZATs to inhibit xylem differentiation

Salinity is a common abiotic stress that hinders plant growth and productivity. In this study, we found that *zat* mutants showed better survival rates compared with Col-0 during salinity treatments. Given the cell death reduction of *zat* mutants in the xylem and root tip, our data suggested that an undamaged root tip and the presence of xylem gap cells played a role in conferring resistance to salt stress in *zat* mutants. We also identified that *ZAT14* was repressed by salinity in vascular tissues but remained unaffected in lateral root cap (LRC) cells. For some ZATs, expression was even increased by salt in epidermal layers (Fig. 6, Supplementary Fig. S5). These data suggest that ZAT downregulation played a key role in modifying vascular development, but the increase in outer cell layers might play another role in modifying cell wall structure in response to stress. Interestingly, the *ZAT14* downstream target *EXPA1* was induced by salinity in both vascular tissues and LRC cells suggesting that *EXPA1* induction in the LRC was not ZAT14 mediated. Similarly, expansin expression increased in *zat* mutants under wild-type conditions (Figs. 5D, and 7A), yet, showed a lower increase during salt stress compared to controls (Fig. 7A), suggesting that ZATs might promote some expansin expression, such as in the LRC where *ZAT14* is not salt-responsive (Fig. 6B). Further work is needed to understand what factors promote *EXPA1* expression in LRC cells and how expansins influence LRC development. Previous studies have shown that root cap-localized genes are essential for halotropic root bending (Galvan-Ampudia et al. 2013; Zheng et al. 2024), suggesting that expansins might be important. We also demonstrated that expansins specifically repress xylem differentiation under salinity conditions (Fig. 8, B and C, Supplementary Fig. S8, B and C), whereas ZATs appear to influence xylem differentiation independently of salinity, suggesting that ZATs have additional downstream targets that regulate xylem development. An outstanding question is how salinity regulates ZATs. We found *ZAT14* expression was oppositely regulated by *VND7* and *PLT2* in root tissues (Figs. 2, C and D and 3F); however, neither of these genes is known to be regulated by salt at the transcriptional level (Augstein and Carlsbecker 2022). Notably, the phosphorylation of PLT2 enhances root growth recovery following the alleviation of salt stress (Hao et al. 2023); however, more work is needed to identify upstream ZAT regulators. Previous studies have shown that many C1-2i subclass members of *ZAT* genes including *AZF1/2* (Sakamoto et al. 2004), *ZAT6* (Liu et al. 2013), *ZAT7* (Ciftci-Yilmaz et al. 2007), *ZAT10* (Sakamoto et al. 2004) are induced by salinity treatment. In contrast, our group of ZATs were negatively regulated in the vascular tissues, and this regulation was highly relevant for reducing xylem differentiation and promoting salinity tolerance. Thus, ZATs integrated developmental cues via PLT and VND transcription factors to opposite regulate xylem differentiation during normal development. During salt stress, their downregulation was important to suppress cell death and promote salinity tolerance, thus highlighting how plants modify their developmental trajectories to better resist stress (Fig. 8H).

## Materials and methods

### Plant material

*Arabidopsis thaliana* Columbia-0 (Col-0) was used as the wild type in this study, except where otherwise indicated. Some reporter and mutant lines used in this study have been previously published, including *pZAT14::3xYFP* (Roszak et al. 2021), *pZAT14L::3xYFP* (Roszak et al. 2021), *35S::XVE>>ZAT14* (Feng et al. 2023), *zat14-1* (Feng et al. 2023), *pWOL::XVE>>ZAT14-mTurq* (Roszak et al. 2021), *35S*::*VND7-GR* plasmid and seeds (Yamaguchi et al. 2010), *vnd6vnd7* (von der Mark et al. 2022), *35S*::*PLT2-GR* (Galinha et al. 2007), *plt1plt2* (Ws-0) (Galinha et al. 2007), *pAHP6::XVE>>PLT2-YFP* (Mähönen et al. 2014), *pEXPA1::n3xGFP* (Ramakrishna et al. 2019), *vnd1,2,3,7* (Ramachandran et al. 2021) and *pRPS5A*  *>*  *GR*  *>*  *EXPA1:mCherry (EXPA1 OE)* (Samalova et al. 2023). *expa10,14,15* was made by crossing *expa10,14* and *expa10,15* (Pacifici et al. 2018). *zat5-1* (SALK_048250) was obtained from the Eurasian *Arabidopsis* Stock Centre (uNASC). *zat5-1zat14-1* was made by crossing *zat5-1* to *zat14-1*. *expa1,10* was made by crossing *expa1* (SALK_010506C) to *expa10* (SALK_053326C). Primers for genotyping are listed in Supplementary Data Set 5. Seeds were surface sterilized with 70% (v/v) ethanol for 10 min, then rinsed with 99.5% (v/v) ethanol. After air-drying, the seeds were either imbibed in sterilized water for VISUAL assays or plated on 1/2 mS medium containing 0.8% (w/v) agar. Seeds were stratified at 4 °C for 2 d before transferred to plant growth cabinet (20–22 °C). Specific growth conditions are outlined in the respective experiment method sections.

### Phylogenetic analysis

The amino acids of C1-2i subclass of the C2H2-type zinc finger protein family (20 genes) were downloaded from The Arabidopsis Information Resource (TAIR). The phylogenetic tree was generated with MEGA11 (Version 11.0.13) using bootstrap method with 1000 replicates, amino acid with *P*-distance method, and partial deletion with 50% site coverage cutoff. The multiple sequence alignment used to generate this phylogeny is provided as a supplementary file labeled Supplementary File 1 and a Newick (.nwk) file labeled Supplementary File 2.

### Generation of transgenic plants

All transformation was performed using the floral dip method (Clough and Bent 1998). To generate *35S::XVE>>ZAT5-YFP*, *35S::XVE>>ZAT14-YFP, 35S::XVE>>ZAT14L-YFP* and *35S::XVE>>ZAT15-YFP*, the open reading frame (ORF) sequence without stop codon was amplified from *Arabidopsis* complementary DNA (cDNA) and cloned into *pDONR221*. Total RNA was isolated using a Roti-Prep RNA MINI Kit and cDNA was synthesized using a First Strand cDNA Synthesis Kit (K1612, Thermo Fisher Scientific). The *p35S::XVE* (Siligato et al. 2016) entry vector carrying the ORF sequence and the *R2P3e-4glyVenusYFP-3AT* (Siligato et al. 2016) vector were combined into the destination vector *pB7M34GW* with a Gateway LR reaction. To generate the *pZAT14::gZAT14-YFP* reporter line, the *p1R4_pZAT14* (Roszak et al. 2021), *pDONR221-ZAT14* (Roszak et al. 2021), and R2P3e-*4glyVenusYFP-3AT* (Siligato et al. 2016) vectors were combined into the *pFR7M34GW* backbone. To generate *pZAT5::3xYFP* and *pZAT15::3xYFP* reporter lines, the promoters of *ZAT5* and *ZAT14* were cloned into the pENTR5′-TOPO vector using a pENTR5′-TOPO TA cloning kit (Invitrogen, catalog nos. K591-10, K591-20, and K5910-00). *pENTR5′-TOPO_pZAT5* or *pENTR5′-TOPO_pZAT15*, *pDONR221-3xYFP* (Roszak et al. 2021) and *R2P3e-nosT2* (Roszak et al. 2021) were combined into a *pFR7M34GW* backbone. To generate CRISPR lines, a single guide RNA (sgRNA) was designed using the Web tool CRISPR-P (http://cbi.hzau.edu.cn/cgi-bin/CRISPR). According to a previously published protocol (Wang et al. 2015), PCR reactions were conducted using gRNA primers, and the *pCBC-DT1T2* plasmid template to amplify the fragments intended for insertion into the *pHEE401E* vector. The CRISPR construct of *ZAT14L* was transformed into the *zat14-1* mutant to obtain *zat14-1zat14 l* double mutants. CRISPR constructs of *ZAT14L* and *ZAT15* were transformed into the *zat5-1zat14-1* background to obtain the *zat* triple (*zatx3*) and quadruple (*zatx4*) mutants. The plasmids were transformed into GV3101 for floral dip. Primary transformants were selected on agar plates containing Hygromycin (50 *µ*g/ml) and genotyped for the inserted or deleted base pairs. Mutants were further confirmed using Sanger sequencing. All primers for cloning and genotyping are listed in Supplementary Data Set 5. CRISPR target information is found in Supplementary Fig. S2, B and C. To generate 35S::*VND7-GR/*Col-0 and 35S::*VND7-GR/zatx3-2* lines, we transformed *35S::VND7-GR* plasmids (Yamaguchi et al. 2010) into the *zatx3-2* background and then performed crosses to either Col-0 or *zatx3-2* to ensure the same transgene was monitored.

### *Arabidopsis* micro-grafting

Seven-day-old seedlings growing in short day condition were used for micrografting following a previously published protocol (Melnyk 2017). For xylem reconnection assays, the roots were removed below hypocotyl junction 5 d after grafting, then 1 *µ*l CFDA (1 mm) was dropped on the wounding site. After 15 min, fluorescence was monitored in the cotyledons as an indication of xylem connectivity.

### Imaging analysis

A Zeiss LSM780 confocal was used for imaging the fluorescence of reporter lines and propidium iodide (PI) staining. Seedlings with or without treatment were mounted in PI solution. Fluorescein diacetate (FDA) combined with PI as a live-death stain for indicating the living cell layers in columella (Huysmans et al. 2018). PI staining was used to indicate the cell death caused by ZATs OE, isoxaben, and salt treatment. For YFP, GFP, and FDA fluorescence imaging, 488 nm excitation and 500–560 nm emission settings were used. 561 nm excitation and 650-690 nm emission were used for PI staining. Lignin was stained with basic fuchsin staining. The images of lignification, xylem gap cells, and swelling cells were observed on a Zeiss Axioscope A1 microscope. A Leica M205 FA stereofluorescent microscope was used for imaging cotyledon vein vascular and ectopic xylem induced in VISUAL assays. A Zeiss LSM800 inverted confocal microscope was used for imaging whole-mount smFISH (WM-smFISH) samples. 561 nm excitation and 570–640 nm emission was used for probes labeled with Quasar570. 405 nm excitation and 400–600 nm emission was used for SCRI Renaissance 2,200 imaging. Pictures of plants were taken using a Nikon D5300 camera. All images were analyzed with Fiji software (version 2.9.0/1.53t).

### Whole-mount smFISH

The roots of 4-day-old *pZAT14::gZAT14-YFP* seedlings was collected for WM-smFISH according to a previous published method (Zhao et al. 2023). Briefly, the root samples were fixed in 4% paraformaldehyde in phosphate-buffered saline (PBS) for 30 min. After washing twice with PBS solution, the samples were transferred into pure methanol and ethanol for 30 min separately. Then, the samples were immersed in ClearSee solution at 4 °C in the dark for 1 d. Samples were washed with Stellaris wash buffer A (Stellaris, Cat.#SMF-WA1-60). The samples were then transferred onto a poly-L slide (Thermo Scientific, Cat.#10219280) and embedded in Hydrogel solution (200 *μ*l of 30% acrylamide–bisacrylamide (29:1) [Bio-Rad, Cat.#1610158], 2 *μ*l of 10% Ammonium Persulfate [APS, Sigma, A-6761], 197 *μ*l of H_2_O and 1 *μ*l of TEMED). Subsequently, they were incubated in a hybridization solution containing the Venus probe at 37 °C overnight in the dark. After washing, the samples were stained with SCRI Renaissance 2200 (ordered from Renaissance Chemicals Ltd, Unit 1 Blackwood Hall Business Park, North Duffield, Selby, UK) solution to mark the cell walls. Finally, the samples were mounted with Vectashield (BioNordika, Cat.# H-1000-10) and imaged on the Zeiss 800 confocal microscope. All probes and chemicals used were the same as previously published (Zhao et al. 2023).

### Vascular cell induction culture system using *Arabidopsis* leaves

VISUAL assay was performed using a previously published method (Kondo et al. 2016). Briefly, stratified seeds were cultured in liquid growth medium (2.2 g L^−1^ MS basal medium, 10 g L^−1^ sucrose and 0.5 g L^−1^ MES, pH 5.7) at 20 °C under continuous light (neutral white fluorescent lamp; 45–55 *μ*mol m^−2^ s^−1^) for 6 d. Then the hypocotyl was cut and the top part with cotyledon was transferred into induction medium (2.2 g L^−1^ MS basal Medium containing 50 g L^−1^ D (+)-Glucose, 1.25 mg L^−1^ 2,4-D, 0.25 mg L^−1^ kinetin and 10 *μ*M bikinin, pH 5.7) at 22 °C under continuous light (neutral white fluorescent lamp; 60–70 *μ*mol m^−2^ s^−1^) for 4 d. The samples were fixed in fixative solution (acetic acid: ethanol = 1:3, v/v) overnight and cleared in clearing solution (chloral hydrate: glycerol: distilled water = 8:1:2, w/v/v). Finally, the auto-fluorescence from xylem secondary cell walls was monitored with a Leica M205 FA stereofluorescent microscope fitted with a UV filter. The area of ectopic xylem was quantified based on autofluorescence intensity using Fiji and normalized to the total cotyledon area.

### ChIP-qPCR assays

Plants of *35S::XVE>>ZAT14-YFP* were grown on 1/2MS plates under LD (16/8 d/night) at 23 °C. For the induction, the stock solution of β-stradiol (Sigma-Aldrich, E8875) was prepared using ethanol as solvent. Six-day-old seedlings were treated either with 8 ml of 10 *μ*M β-estradiol per plate, or 8 ml of control solution. After an induction period of 6 h, approximately 1.0 g of tissue was collected per sample and frozen in liquid nitrogen.

ChIP was performed as described in Kaufmann et al. (2010) with the following changes: samples were fixed in 1% formaldehyde for 20 min after grinding, sonication was performed using a Covaris M220 system (conditions: Peak Incident Power 75, Duty Factor 10, Cycles per burst 200, time 900 s), the incubation time with the antibody (Anti-GFP ab290; AbCam) was increased to overnight (16 h at 4 °C), the incubation time with protein-A agarose beads was increased to 6 h, and the purification of DNA after decrosslinking was performed with a MinElute Reaction Cleanup Kit (Qiagen). For the *EXPA1* locus, primers were designed within 1500 bp upstream of the transcription start site (TSS). For the *EXPA10*, *EXPA14*, *EXPA15*, and *PRX71* loci, primers were designed to flank regions containing the previously published ZAT-binding consensus sequence, AGT(N_1_₋_3_)ACT (Franco-Zorrilla et al. 2014). Percentage of input was calculated using 3 biological replicates for the treatment and control, and the Mann–Whitney *U* test was used for statistical differences.

### Reverse transcription-quantitative PCR assays

The root samples were collected for RNA extraction using a Roti-Prep RNA MINI Kit, and cDNA was synthesized using a First Strand cDNA Synthesis Kit (K1612, Thermo Fisher Scientific). 2× Maxima SYBR Green qPCR/ROX Master Mix (Thermo Fisher Scientific) was used for the reaction and the reactions was performed using a Bio-Rad CFX96 qPCR machine. The *Arabidopsis PROTEIN PHOSPHATASE 2A* (*PPA2*) was used as reference gene. The results were analyzed using the 2^−ΔΔCT^ method. All primers are listed in Supplementary Data Set 5.

### Salt tolerance assay

The sterilized seeds were plated and grown horizontally on 25 mm pore Sefar Nitex 03–25/19 mesh on 1/2 mS medium (0.8% agar, pH 5.7). Three days after growing in long day condition (16/8 h light/dark, ∼110 μmol m^−2^ s^−1^, 22 °C, chamber), the seedlings with mesh were transferred to medium with 200 mm NaCl, a concentration that typically results in the death of *Arabidopsis* seedling, for 5 d. The seedlings showing green were counted as living plants and results shown with survival rates. For seedlings survival assay under lower salt concentration, 3-d-old seedling were grown along with wild-type on medium containing 140 mm NaCl for 10 d and then counted the survival score of the plants by the color of the cotyledons (white vs green or pale green). Survival score was calculated by assigning plants with white cotyledons a score of 1, pale green 3 and green 5. These scores were multiplied and then divided by the number of analyzed plants. For VISUAL assays, we used 100 mm NaCl. For salinity-induced cell death in roots, xylem gap observations and salt affected gene expression analyses, 140 mm NaCl was used.

### Quantification of xylem gap cells

For xylem gap cells observation, roots were mounted in a chloral hydrate solution (8:3:1, chloral hydrate:water:glycerol, w/v/v) and visualized using a Zeiss Axioscope A1 microscope equipped with differential interference contrast (DIC) optics at ×40 magnification, as previously described (Augstein and Carlsbecker 2022). The frequency of plants exhibiting the protoxylem or metaxylem gap phenotype was recorded, and the number of gaps per root was quantified.

### RNA-sequencing analysis

Five-day-old seedlings expressing *35S::XVE>>ZAT14-YFP* were induced with 5 *μ*M estradiol for 8, 16, and 24 h. Seedlings were treated with ethanol as a control. For the generation of libraries of *zatx3* and *zatx4* libraries, five-day-old Col-0 wild type seedlings were used as a control. The roots were collected for RNA extraction. The total RNA was extracted using RNA extraction using a Roti-Prep RNA MINI Kit. One microgram RNA per sample was used for preparing the library. NEBNext Poly(A) mRNA Magnetic Isolation Module (NEB number E7490S), NEBNext Ultra Directional RNA Library Prep Kit for Illumina (NEB number E7760S), and NEBNext Multiplex Oligos for Illumina (NEB number E7600S) were used for library construction. Libraries were sequenced at Novogene on the Illumina NovaSeq 6000 system with PE150. The raw reads were quality-filtered using fastp (version 0.20.0). The cleaned reads were mapped to the *Arabidopsis* reference genome TAIR10 using hisat2 (version 2.2.2). Gene abundance was quantified with HTSeq (version 0.12.4). Transcript levels were then analyzed using the DESeq2 R package (version 3.13) to identify differentially expressed genes (DEGs), with a q-value threshold of < 0.05. Gene ontology (GO) enrichment analysis was conducted using the ClusterProfiler R package (version 3.14.0), with an adjusted *P*-value < 0.001 and the number of genes > 10. The lists of DEGs and GO annotations are provided in Supplementary Data Sets S1 and S3. GO analysis for genes differentially expressed in *zat* mutants or *ZAT14-OE* and upon salt was done using pantherdb.org.

### Quantification and statistical analysis

Area quantifications were performed using Fiji. Statistical analysis methods are specified in the figure legends. Student's *t*-test, one-way ANOVA was conducted using GraphPad Prism 10 (Version 10.2.1). The Mann–Whitney *U* test analysis for ChIP-qPCR assay was performed in R (Version 2023.03.1), for treatment (+estradiol) versus control (-estradiol) on each tested fragment, using a *P*-value of <0.05 as cutoff. The following settings were used: Mann–Whitney *U* test (treatment, control, alternative = “greater”, correct = FALSE, exact = FALSE). The multiple Fisher's exact test was also performed in R (Version 2023.03.1). The analyzed results are listed in Supplementary Data Set 6.

### Accession numbers

*ZAT5* (AT2G28200), *ZAT14* (AT5G03510), *ZAT14L* (AT5G04390), *ZAT15* (AT3G10470), *VND1* (AT2G18060), *VND2* (AT4G36160), *VND3* (AT5G66300), *VND6* (AT5G62380), *VND7* (AT1G71930), *XCP1* (AT4G35350), *XCP2* (AT1G20850), *PLT1* (AT3G20840), *PLT2* (AT1G51190), *EXPA1* (AT1G69530), *EXPA8* (AT2G40610), *EXPA9* (AT5G02260), *EXPA10* (AT1G26770), *EXPA12* (AT3G15370), *EXPA14* (AT5G56320), *EXPA15* (AT2G03090), *EXPA17* (AT4G01630), *EXPA18* (AT1G62980), *EXLB1* (AT4G17030), *EXPB1* (AT2G20750), *PRX71* (AT5G64120), *SMB* (AT1G79580) and *BRN2* (AT4G10350).

## Supplementary Material

koaf271_Supplementary_Data

## Data Availability

The transcriptomic data that support the findings of this study have been deposited in the NCBI Bioproject database under the accession number: PRJNA1123729 (https://www.ncbi.nlm.nih.gov/sra/PRJNA1123729). This study did not generate original code.

## Supplementary data

The following materials are available in the online version of this article.

**Supplementary Figure S1.**  *ZAT* expression patterns (Supports Fig. 1).

**Supplementary Figure S2.** PLT2 regulates ZATs and a description of the ZAT mutant alleles (Supports Fig. 2).

**Supplementary Figure S3.** ZATs are involved in xylem differentiation (Supports Figs. 3 and 4).

**Supplementary Figure S4.**  *ZAT14* affects cell wall-related processes (Supports Fig. 5).

**Supplementary Figure S5.** Transcriptional reporters of *ZAT14* and its homologs upon salt treatment (Supports Fig. 6).

**Supplementary Figure S6.** Analysis of genes differentially expressed in *ZAT14OE*/*zatx4* and in response to salt (Supports Fig. 6).

**Supplementary Figure S7.** ZAT14 directly binds to cell wall-related genes (Supports Fig. 7).

**Supplementary Figure S8.** Salinity promotes cell wall damage and inhibits xylem differentiation (Supports Fig. 8).

**Supplementary Data Set 1.** List of genes differentially expressed in *ZAT14 OE* and *zat* mutants.

**Supplementary Data Set 2.** List of genes downregulated in *ZAT14 OE* and upregulated in *zat* mutants.

**Supplementary Data Set 3.** GO-enrichment of genes downregulated in *ZAT14 OE* and upregulated in *zat* mutants.

**Supplementary Data Set 4.** Overlapped genes differentially expressed in *ZAT14 OE*/*zatx4* in response to salt.

**Supplementary Data Set 5.** Primer list for cloning and CRISPR constructs.

**Supplementary Data Set 6.** Statistical analysis results.

**Supplementary Data File 1.** A FASTA file of C1-2i subclass of the C2H2-type zinc finger protein family.

**Supplementary Data File 2.** A Newick file of C1-2i subclass of the C2H2-type zinc finger protein family.

## Dive Curated Terms

The following phenotypic, genotypic, and functional terms are of significance to the work described in this paper:

ZAT Gramene: AT2G46800
ZAT Araport: AT2G46800

AZF2 Gramene: AT3G19580
AZF2 Araport: AT3G19580

ZAT7 Gramene: AT3G46090
ZAT7 Araport: AT3G46090

APL Gramene: ostta07g03440
APL Araport: ostta07g03440

AZF1 Gramene: AT5G67450
AZF1 Araport: AT5G67450

ZAT6 Gramene: AT5G04340
ZAT6 Araport: AT5G04340

CESA1 Gramene: Os05g0176100
CESA1 Araport: Os05g0176100

CESA4 Gramene: AT5G44030
CESA4 Araport: AT5G44030

CESA5 Gramene: AT5G09870
CESA5 Araport: AT5G09870

GFP Gramene: green fluorescent protein
GFP Araport: green fluorescent protein

PPa2 Gramene: AT2G18230
PPa2 Araport: AT2G18230
