# Supplementary material for: The ZAT14 family promotes cell death and regulates expansins to affect xylem formation and salt tolerance in Arabidopsis
Source: Plant Cell. 2025 Nov 13;37(12):koaf271. doi: 10.1093/plcell/koaf271 (PMC12702618; doi:10.1093/plcell/koaf271)
Supplement: koaf271_Supplementary_Data [file koaf271_supplementary_data.zip › Supplementary_Data.pdf]

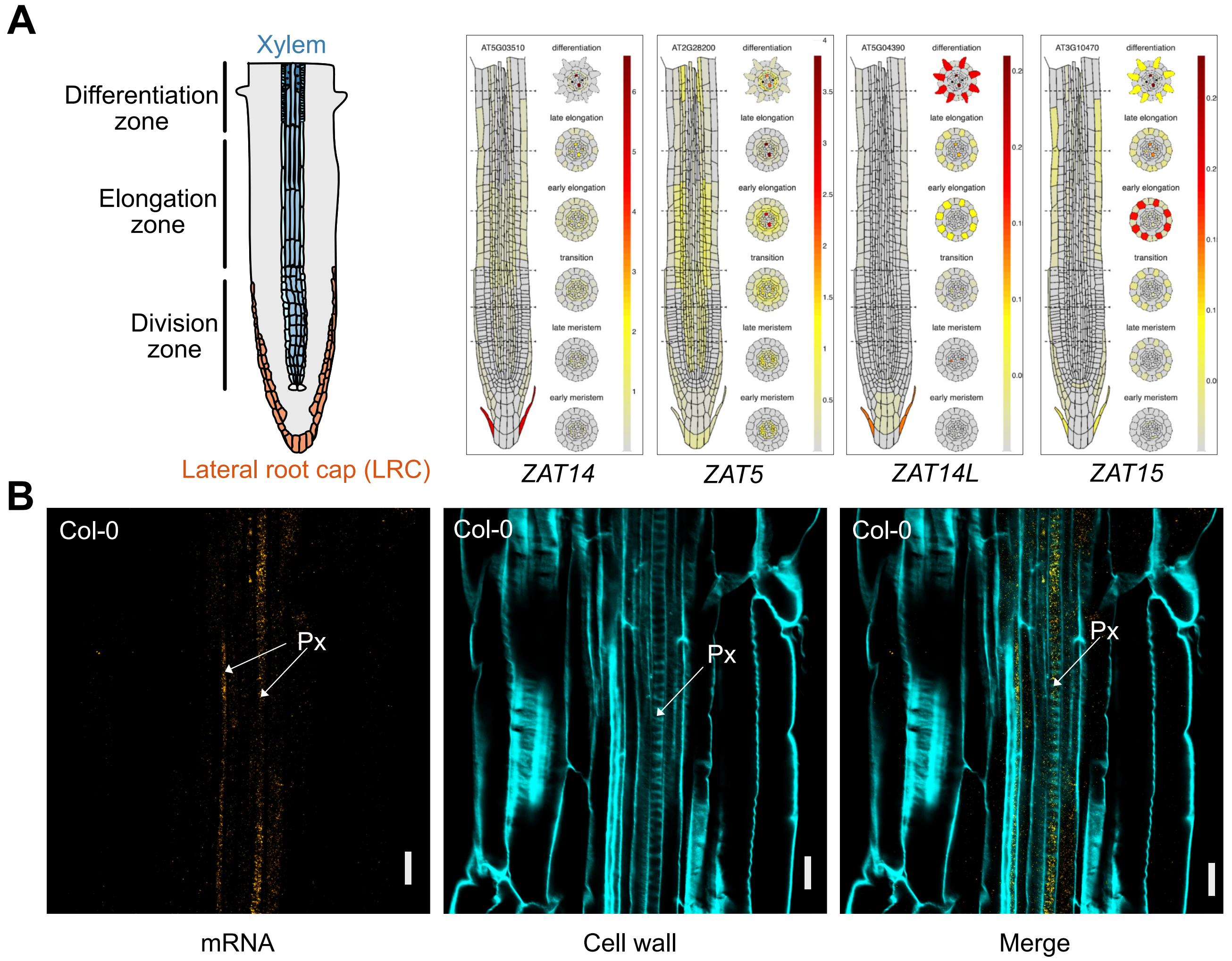

Supplementary Figure S1. *ZAT* expression patterns (Supports Figure 1). **A**) Schematics showing an overview of different *Arabidopsis* root tissues. Different root zones, xylem and lateral root cap (LRC) are shown. Right panels show ZAT expression patterns, data are from https://rootcellatlas.org/. **B)** Whole-mount smFISH (WM-smFISH) on endogenous *ZAT14* showed mRNA (yellow) localized in developing protoxylem cells of 5 days old seedlings. mRNA molecules were detected with *ZAT14* probes, cell walls were stained with Renaissance 2200. Px, protoxylem. Scale bars, 10 µm.

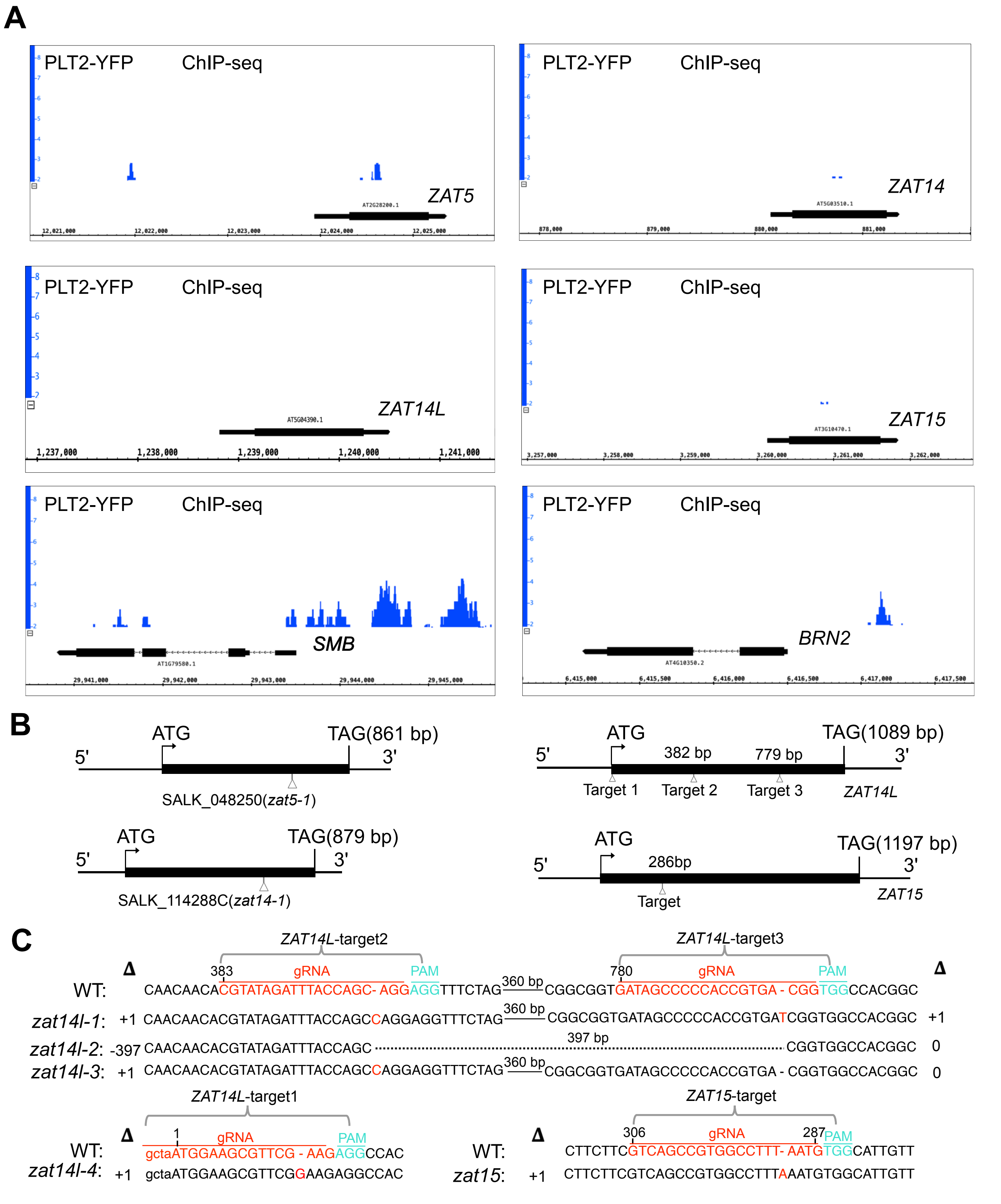

Supplementary Figure S2. PLT2 regulates *ZATs* and a description of the ZAT mutants alleles (Supports Figure 2). A) Integrated Genome Browser screen shots showing ChIP-seq for PLT2-YFP at *ZAT5*, *ZAT14*, *ZAT14L*, *SOMBRERO* (*SMB*) and *BEARSKIN 2* (*BRN2*) locus. The ChIP-seq data were previously reported (Santuari et al., 2016). **B)** Schematic illustration of T-DNA mutants of *ZAT5* and *ZAT14* and CRISPR mutation sites of *ZAT14L* and *ZAT15*. **C)** CRISPR mutated sequences of *ZAT14L* and *ZAT15*. *zatx3-1*: *zat14-1zat5-1zat14l-1*. *zatx3-2*: *zat14-1zat5-1zat14l-2*. *zatx3-3*: *zat14-1zat5-1zat14l-3*. *zatx4*: *zat14-1zat5-1zat14l-3zat15*.

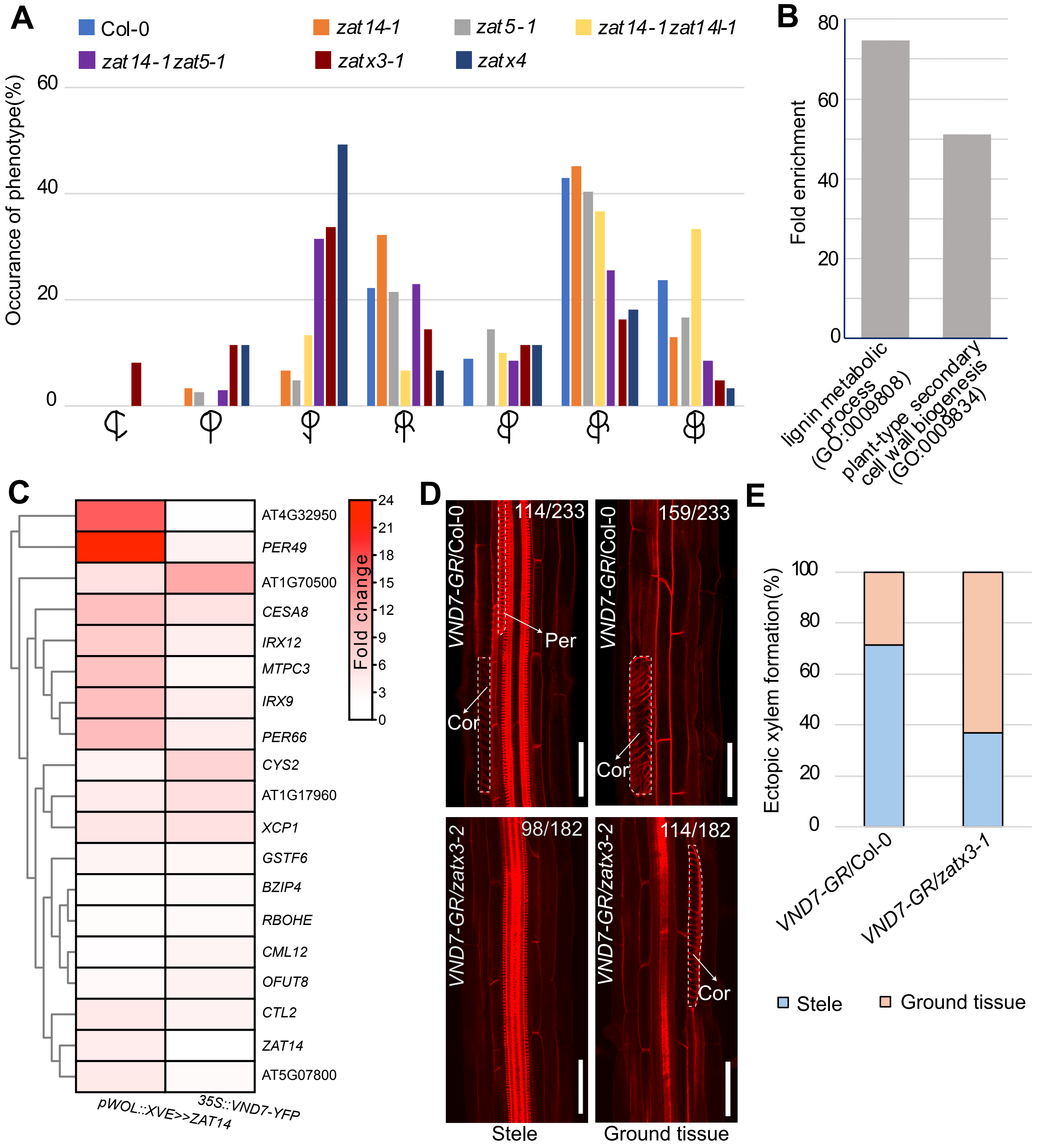

Supplementary Figure S3. ZATs are involved in xylem differentiation (Supports Figures 3 and 4). A) Occurrence of vein vascular phenotypes. Col-0 (n=165), *zat14-1* (n=62), *zat5-1* (n=84), *zat14-1zat14l-1-4* (n=59), *zat14-1zat5-1* (n= 69), *zatx3-1* (81), *zatx4* (61). **B)** Gene Ontology (GO) enrichment analysis of genes upregulated by both *VND7* and *ZAT14*. **C)** Heatmap showing the expression of the 19 overlapping genes from Figure 3H. **D)** *VND7-GR* induces ectopic xylem formation in Col-0 and *zatx3-2* backgrounds. 5-day-old seedlings were induced with 30 µM DEX for 48 h. *VND7-GR* was transferred into the Col-0 (*VND7-GR*/Col-0) and *zatx3-2* (*VND7-GR/zatx3-2*) backgrounds. 10 seedlings were examined for each genotype. In *VND7-GR*/Col-0, ectopic xylem predominantly formed within the vascular tissue, whereas in *VND7-GR/zatx3-2*, it primarily formed outside the vascular tissue. The xylem was stained with basic fuchsin. Per, pericycle. Cor, cortex. one slide from z stacks showed the different cell types. The slide number was labelled on the images. Ectopic xylem regions are outlined with dashed lines. Scale bars, 50 µm. **E)** Quantification of ectopic xylem formation. 10 roots were quantified for each genotype.

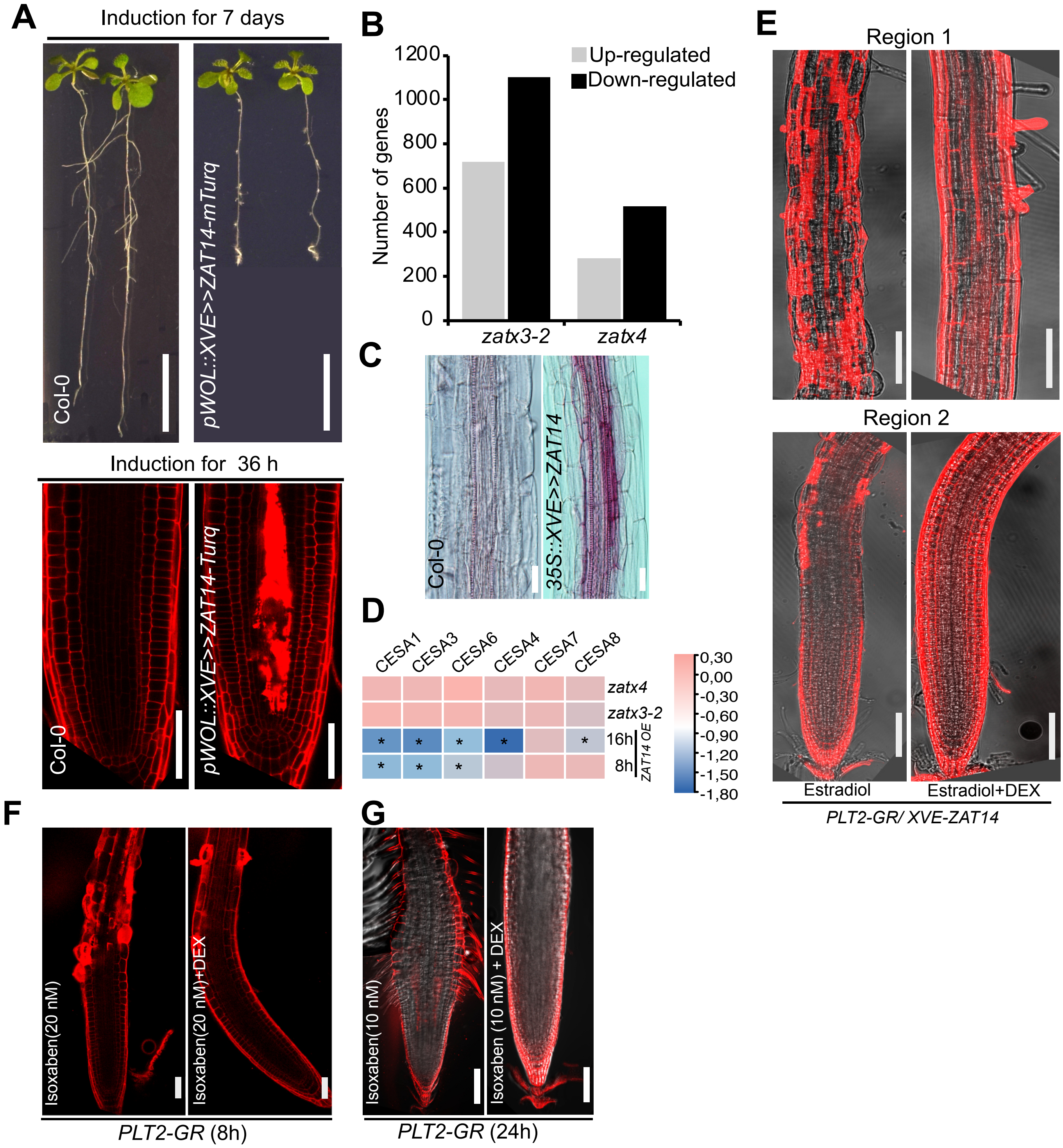

Supplementary Figure S4. *ZAT14* affects cell wall-related processes (Supports Figure 5). A) Ectopic overexpression of *ZAT14* promotes cell death in root meristematic cells. 5-day-old wild type or *pWOL::XVE>>ZAT14-mTurq* seedlings were induced with 10 µM estradiol. The cut off corner results from cropping. Scale bars, 1 cm for upper panel, 50 µm for lower panel. Red is propidium iodide staining. **B)** Number of differentially expressed genes (DEGs) in the RNA-seq data of *zat* mutants. **C)** *35S::XVE>>ZAT14* promotes lignification (red signal, stained with basic fuchsin). 10 µM estradiol induction for 24 h with 5-day-old seedlings, then recovered on 1/2 MS for 3 days. Scale bars, 50 µm. **D)** Primary CESA genes (*CESA1*, *CESA3* and *CESA6*) and secondary CESA genes (*CESA4*, *CESA7* and *CESA8*) expression. 8 h and 16 h indicate two time points of *ZAT14 OE* induction. **E)** *PLT2* rescues *35S::XVE>>ZAT14*-induced cell wall damage and cell swelling. 5 µM estradiol and 10 µM DEX were treated on 5-day-old F1 seedlings for 24 h. Red is propidium iodide staining. The cut off corner results from cropping. Scale bars, 100 µm. **F)** *PLT2* inhibits isoxaben-induced cell wall damage. 5-day-old seedlings were treated with isoxaben (20 nM) or isoxaben (20 nM) + DEX (10 µM) for 8 h, seedlings were treated with Ethanol or DEX (10 µM) for 4 h before transferring to medium with isoxaben. Red is propidium iodide staining. Scale bars, 50 µm. **G)** *PLT2* rescues isoxaben-induced cell wall swelling. Isoxaben 10 nM. DEX, 20 µM. Isoxaben and DEX treatments were for 24 h. Red is propidium iodide staining. Scale bars, 100 µm.

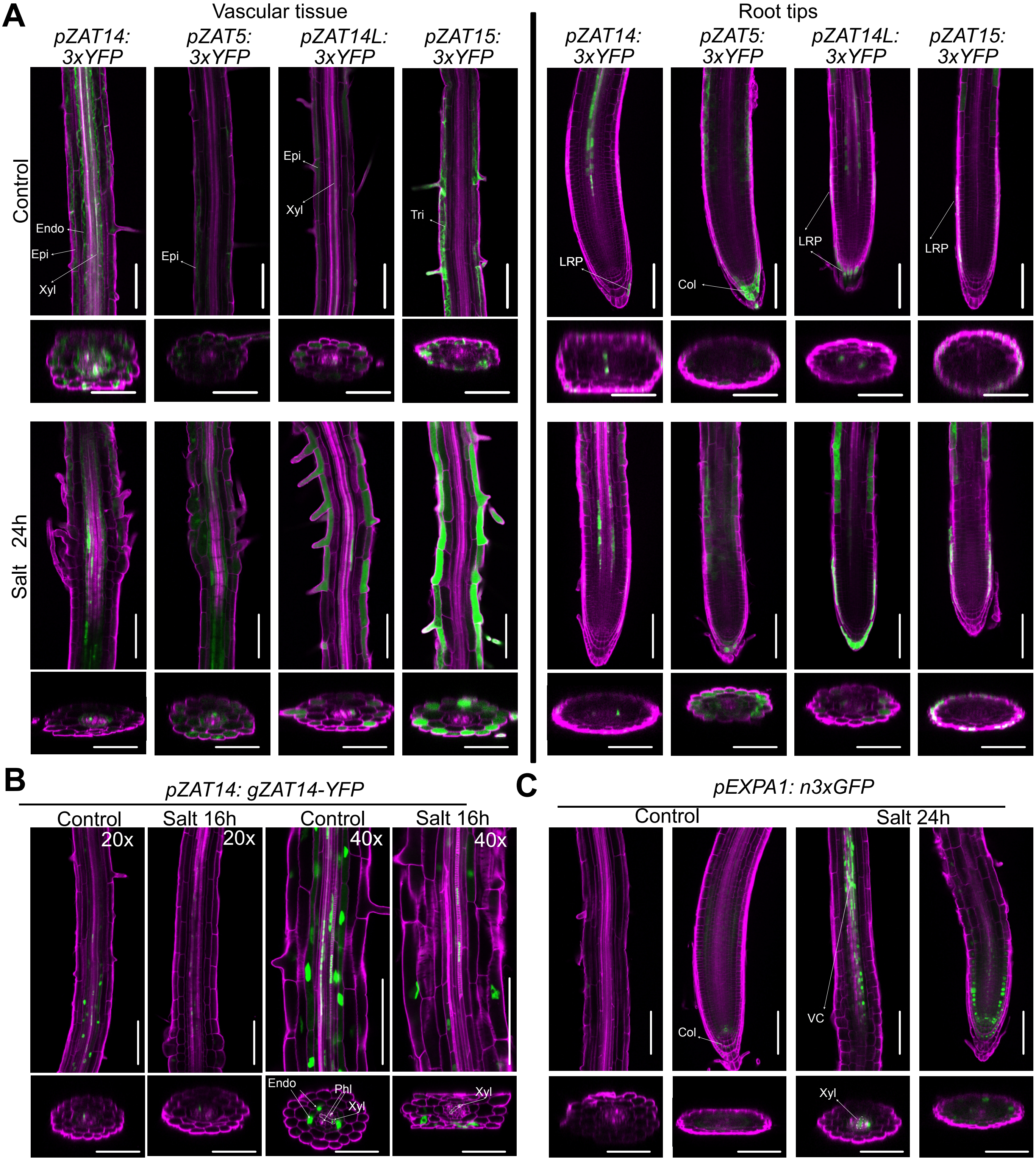

Supplementary Figure S5. Transcriptional reporters of *ZAT14* and its homologs upon salt treatment.(Supports Figure 6). A) Transcriptional reporter of *ZAT14* and its homologs 24 h after salt treatment (140 mM NaCl). **B)** *pZAT14::gZAT14-YFP* levels (green) in response to salt (140 mM NaCl) at 16 h after treatment. Longitudinal and cross sections were shown with 20x and 40x objectives. **C)** The *pEXPA1:n3xGFP* reporter showed increased fluorescence 24 h after salt treatment (140 mM NaCl). Longitudinal and cross sections were shown with 20x in (A and C). Scale bars, 100 µm in longitudinal sections and 50 µm in cross sections. The fluorescent cell types are annotated in the related images of (A-C). Epi, epidermis. Endo, endodermis. Vas, vasculature. VC, vascular cambium. Phl, phloem. Xyl, xylem. Tri, trichoblast. LRC, lateral root cap. Col, columella.

A

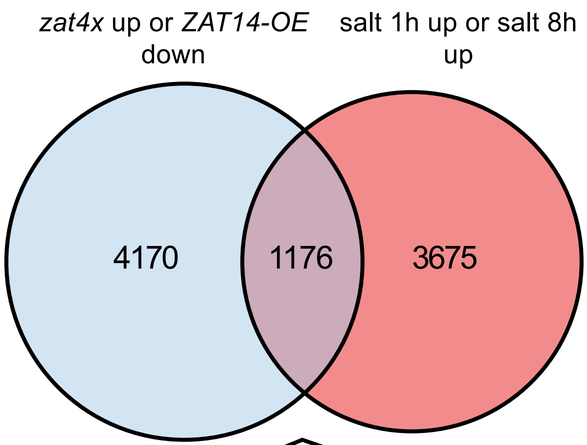

GO biological process complete	Fold Enrichment	P-value
glutamine metabolic process (GO:0006541)	9.76	8.68E-03
disaccharide biosynthetic process (GO:0046351)	5.77	4.86E-02
toxin catabolic process (GO:0009407)	5.15	4.85E-02
disaccharide metabolic process (GO:0005984)	4.86	3.68E-04
plant-type secondary cell wall biogenesis (GO:0009834)	4.82	6.15E-03
toxin metabolic process (GO:0009404)	4.74	1.79E-02
oligosaccharide metabolic process (GO:0009311)	4.41	1.51E-03
monocarboxylic acid biosynthetic process (GO:0072330)	4.41	6.91E-11
auxin polar transport (GO:0009926)	4.34	2.07E-02
fatty acid biosynthetic process (GO:0006633)	3.84	4.06E-05
detoxification (GO:0098754)	3.66	5.71E-05
response to toxic substance (GO:0009636)	3.61	1.13E-05
monocarboxylic acid metabolic process (GO:0032787)	3.44	2.65E-14
cell wall macromolecule metabolic process (GO:0044036)	3.26	3.27E-04
carbohydrate biosynthetic process (GO:0016051)	3.2	5.25E-08
response to water deprivation (GO:0009414)	3.15	8.26E-11
response to water (GO:0009415)	3.11	8.44E-11
polysaccharide biosynthetic process (GO:0000271)	3.08	1.35E-04
fatty acid metabolic process (GO:0006631)	3.04	3.87E-05
organic acid biosynthetic process (GO:0016053)	2.98	2.01E-10
carboxylic acid biosynthetic process (GO:0046394)	2.98	2.01E-10
response to nutrient levels (GO:0031667)	2.96	7.52E-05
cell wall polysaccharide metabolic process (GO:0010383)	2.93	4.77E-02
response to acid chemical (GO:0001101)	2.89	1.91E-09
glucan metabolic process (GO:0044042)	2.84	4.91E-03
secondary metabolic process (GO:0019748)	2.82	1.40E-02
response to carbohydrate (GO:0009743)	2.8	2.47E-02
carboxylic acid metabolic process (GO:0019752)	2.76	1.15E-16
cell wall organization or biogenesis (GO:0071554)	2.72	2.83E-09
plant-type cell wall organization or biogenesis (GO:0071669)	2.7	7.07E-04
polysaccharide metabolic process (GO:0005976)	2.66	9.67E-07
carbohydrate metabolic process (GO:0005975)	2.61	7.41E-16
oxoacid metabolic process (GO:0043436)	2.59	1.36E-15
organic acid metabolic process (GO:0006082)	2.59	1.47E-15
small molecule biosynthetic process (GO:0044283)	2.57	4.90E-09
cell wall organization (GO:0071555)	2.55	3.59E-03
response to osmotic stress (GO:0006970)	2.48	2.38E-07
response to abscisic acid (GO:0009737)	2.41	7.98E-06

B

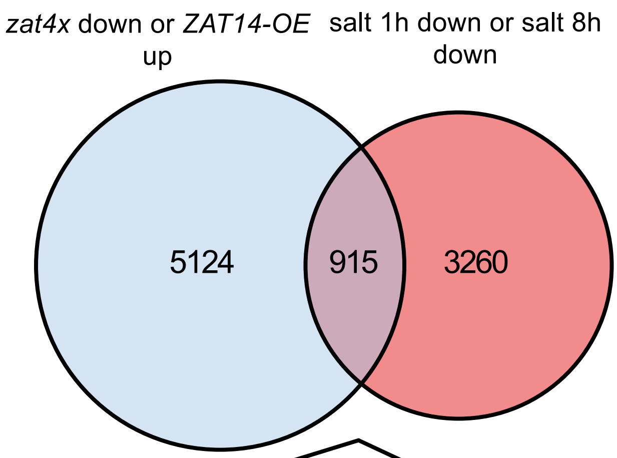

GO biological process complete	Fold Enrichment	P-value
hydrogen sulfide biosynthetic process (GO:0070814)	19.12	5.28E-03
hydrogen sulfide metabolic process (GO:0070813)	17	1.16E-02
defense response by callose deposition in cell wall (GO:0052544)	11.47	2.01E-02
defense response by cell wall thickening (GO:0052482)	10.8	3.02E-02
S-glycoside biosynthetic process (GO:0016144)	9.25	1.82E-06
glucosinolate biosynthetic process (GO:0019761)	9.25	1.82E-06
glycosyl compound biosynthetic process (GO:1901659)	7.95	1.43E-05
S-glycoside metabolic process (GO:0016143)	4.56	5.04E-04
glucosinolate metabolic process (GO:0019760)	4.56	5.04E-04
sulfur compound biosynthetic process (GO:0044272)	4.49	1.12E-05
glycosyl compound metabolic process (GO:1901657)	3.6	7.86E-03
sulfur compound metabolic process (GO:0006790)	2.74	1.29E-03
cellular response to lipid (GO:0071396)	2.48	3.03E-02
cellular response to chemical stimulus (GO:0070887)	1.81	1.10E-02
response to endogenous stimulus (GO:0009719)	1.7	1.61E-02
response to oxygen-containing compound (GO:1901700)	1.69	4.78E-03
response to hormone (GO:0009725)	1.69	2.21E-02
response to chemical (GO:0042221)	1.67	1.10E-06
cellular response to stimulus (GO:0051716)	1.58	1.28E-04
localization (GO:0051179)	1.48	3.83E-02
response to stimulus (GO:0050896)	1.47	3.98E-09
response to stress (GO:0006950)	1.46	1.33E-03
Unclassified (UNCLASSIFIED)	1.19	0.00E+00
cellular process (GO:0009987)	1.19	1.22E-02

Supplementary Figure S6. Analysis of genes differentially expressed in *ZAT14OE*/*zatx4* and in response to salt (Supports Figure 6). A, B) Venn diagrams and GO-term analyses of overlapping genes: (A) down-regulated genes by *ZAT14* and up-regulated genes by salt treatment, and (B) up-regulated genes by *ZAT14* and down-regulated genes by salt treatment.

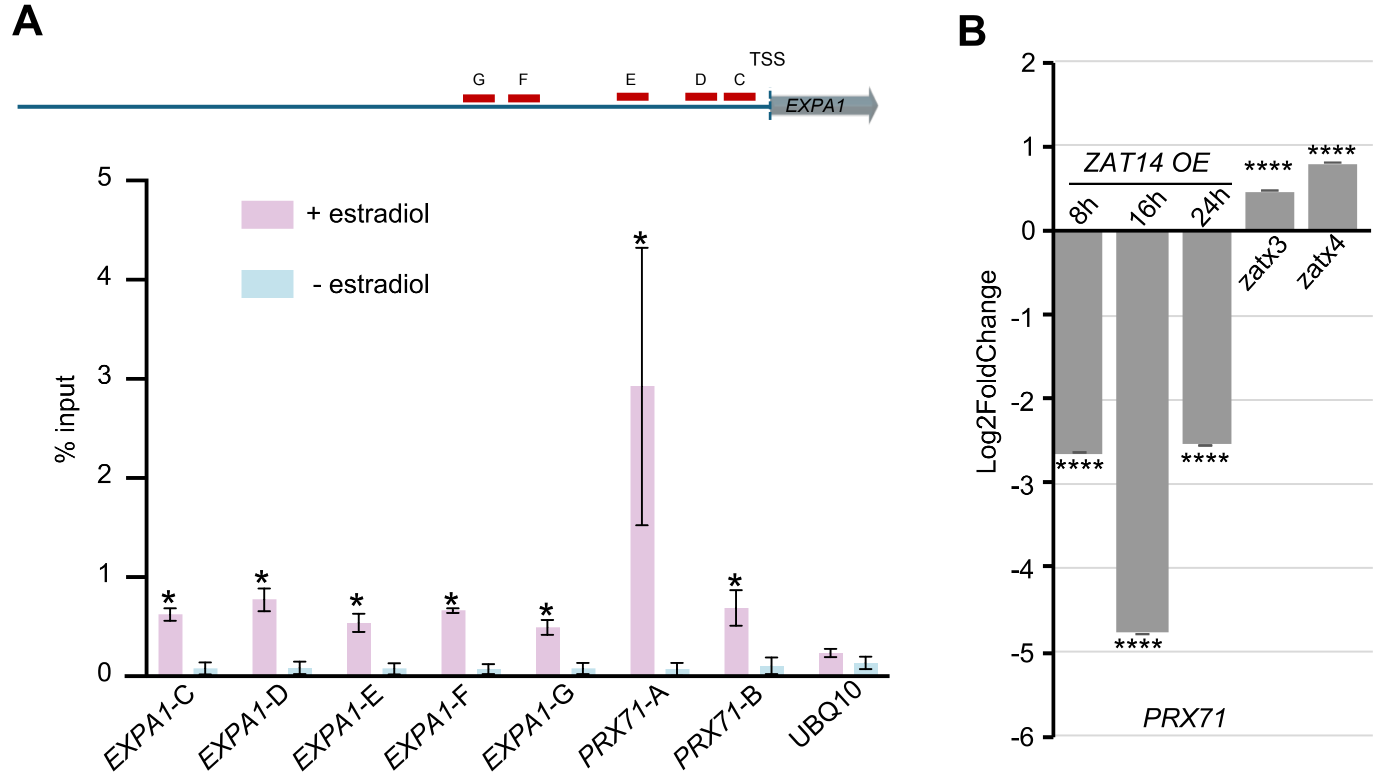

Supplementary Figure S7. ZAT14 directly binds to cell wall-related genes (Supports Figure 7). A) ChIP-qPCR showed ZAT14 directly binds the promoter of *EXPA1* and *PRX71*, *UBQ10* was as a negative control. Three replicates. Means ± SE. The Mann-Whitney U test was used for statistical differences, * $p<0.05$. **B)** RNA-seq data showed *PRX71* was downregulated by *ZAT14 OE* at 8 h, 16 h and 24 h induction time points, and upregulated in *zat* mutants. Data is shown in Supplementary Data Set S1. ****$p<0.0001$.

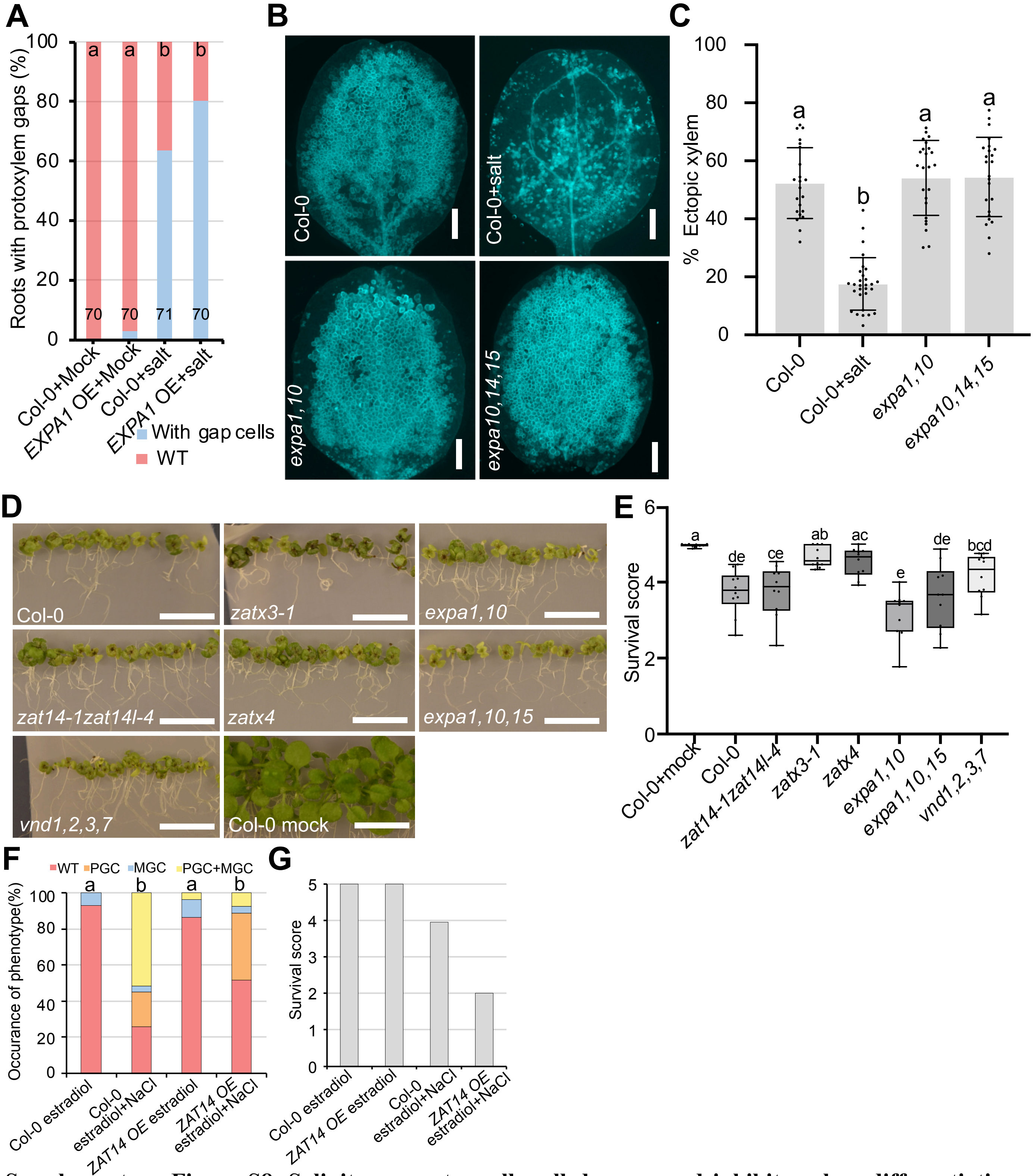

Supplementary Figure S8. Salinity promotes cell wall damage and inhibits xylem differentiation (Supports Figure 8). A) Xylem gap phenotype after salt treatment. Three days old seedlings were treated with 140 mM NaCl for 3 days. Numbers indicate n; letters indicate statistical significance with multiple Fisher's exact test and Benjamini–Hochberg (BH) correction. **B)** VISUAL assay of Col-0 with or without salt treatment, *expa1,10* and *expa10,14,15*. Scale bars, 500 µm. **C)** Quantification of ectopic xylem in Col-0 (n=22), Col-0+100 mM NaCl (n=28), *expa1,10* (n=24), *expa10,14,15* (n=24). One-way ANOVA followed by Tukey's multiple comparisons test. Letters indicate statistically significant differences. **D)** Salinity tolerance of mutants. 3-day-old seedlings were transferred to medium with NaCl (140 mM) for 10 days. Scale bars, 1 cm. **E)** Survival score of plants on 140 mM salt. Means ± SD, five replicates with Col-0 mock (no salt treatment, n=145), Col-0 (n=134), *zat14-1zat14l-4* (n=134), *zatx3-1* (n=138), *zatx4* (n=131), *expa1,10* (n=131), *expa1,10,15* (n=141), *vnd1,2,3,7* (n=148). Letters indicate statistically significant differences. One-way ANOVA followed by Tukey's multiple comparisons test. **F)** Xylem gap phenotype after salt treatment. 3 days old Col-0 and *ZAT14 OE* seedlings germinated on medium containing 10nM estradiol, then transferred to medium containing 10nM estradiol+140 mM NaCl or 10 nM estradiol for 3 days. WT, wild type xylem phenotype. PGC, protoxylem gap cells. MGC, metaxylem gap cells. The statistical analysis was compared between WT and xylem gap (PGC, MGC and PGC+MGC) phenotypes. Letters indicate statistical significance with multiple Fisher's exact test and Benjamini–Hochberg (BH) correction. **G)** Survival score of plants on 140 mM salt. 3 days old seedlings germinated on medium containing 10nM estradiol, then transferred to medium containing 10nM estradiol+140 mM NaCl or 10 nM estradiol for 4 days.
